# Effect of Genotype, Year, and Their Interaction on the Accumulation of Bioactive Compounds and the Antioxidant Activity in Industrial Hemp (*Cannabis sativa* L.) Inflorescences

**DOI:** 10.3390/ijms24108969

**Published:** 2023-05-18

**Authors:** Romina Beleggia, Valeria Menga, Flavia Fulvio, Clara Fares, Daniela Trono

**Affiliations:** 1 Council for Agricultural Research and Economics (CREA), Research Centre for Cereal and Industrial Crops, S.S. 673, Km 25,200, 71122 Foggia, Italy; valeria.menga@crea.gov.it (V.M.); clara.fares@crea.gov.it (C.F.); daniela.trono@crea.gov.it (D.T.); 2 Council for Agricultural Research and Economics (CREA), Research Centre for Cereal and Industrial Crops, Via di Corticella, 133, 40128 Bologna, Italy; flavia.fulvio@crea.gov.it

**Keywords:** hemp, phenolic compounds, terpenes, cannabinoids, antioxidant activity, genotype, cropping year

## Abstract

The phytochemical content and the antioxidant activity in the inflorescences of six industrial hemp (*Cannabis sativa* L.) genotypes, four monoecious (Codimono, Carmaleonte, Futura 75, and Santhica 27), and two dioecious (Fibrante and Carmagnola Selezionata), were assessed for three consecutive years from 2018 to 2020. The total phenolic content, total flavonoid content, and antioxidant activity were determined by spectrophotometric measurements, whereas HPLC and GC/MS were used to identify and quantify the phenolic compounds, terpenes, cannabinoids, tocopherols, and phytosterols. All the measured traits were significantly affected by genotype (G), cropping year (Y), and their interaction (G × Y), although the Y effect prevailed as a source of variation, ranging from 50.1% to 88.5% for all the metabolites except cannabinoids, which were equally affected by G, Y, and G × Y interaction (33.9%, 36.5%, and 21.4%, respectively). The dioecious genotypes presented a more constant performance over the three years compared to the monoecious genotypes, with the highest and most stable phytochemical content observed in the inflorescences of Fibrante, which was characterized by the highest levels of cannabidiol, α-humulene and β-caryophyllene, which may confer on the inflorescences of this genotype a great economic value due to the important pharmacological properties of these metabolites. Conversely, the inflorescences of Santhica 27 were characterized by the lowest accumulation of phytochemicals over the cropping years, with the notable exception of cannabigerol, a cannabinoid that exhibits a wide range of biological activities, which was found at its highest level in this genotype. Overall, these findings can be used by breeders in future programs aimed at the selection of new hemp genotypes with improved levels of phytochemicals in their inflorescences, which can provide better health and industrial benefits.

## 1. Introduction

*Cannabis sativa* L. is an herbaceous plant with an annual cycle that belongs to the *Cannabaceae* family [1]. Its domestication dates back to 6000–8000 years ago in Central and Eastern Asia [2]. It is spread worldwide and is considered one of the oldest crops employed for multi-purpose applications, also thanks to its climatic and territorial adaptability [3]. *C. sativa*, naturally, is a dioecious crop, but monoecious cultivars have also been obtained through breeding efforts [4].

Based on the content of the psychotropic cannabinoid Δ9-tetrahydrocannabinol (THC) and its ratio with the non-psychotropic cannabinoid cannabidiol (CBD), three different chemotypes of *C. sativa* have been identified: chemotype I or drug-type with THC > 0.3% of the inflorescence dry weight and CBD < 0.5% (THC/CBD ratio >> 1), chemotype II or intermediate-type with both THC and CBD as the predominant cannabinoids (THC/CBD ratio between 0.5 and 1), and chemotype III or fiber-type with high CBD and very low THC content (THC/CBD << 1) [5]. Subsequently, two other fiber-type chemotypes have been identified: chemotype IV, which contains the non-psychotropic cannabinoid cannabigerol (CBG) as the main cannabinoid (>0.3%) but also CBD (<0.5%) [6], and chemotype V, which contains undetectable amounts of cannabinoids [7]. In the European Union (EU), only the cultivation of hemp cultivars registered in the EU Common Catalogue of Varieties of Agricultural Plant Species [8] and with a THC content below 0.3% is permitted [9].

Hemp is a crop with many applications and uses. The most common harvestable products of hemp are fibers from the straw and seeds [10]. Fibers are used in the textile, paper, biofuel, biodegradable plastics, and construction industries [11], whereas seeds, with their high nutritional value as a source of proteins, unsaturated fatty acids, antioxidants, and minerals, are widely employed as food and supplement ingredients [12]; sprouts from hemp seeds also represent an attractive functional food due to their high antioxidant properties [13].

By contrast, hemp inflorescences are traditionally recognized as waste products for the fiber and seed industries. However, more recently, evidence is accumulating that suggests that the inflorescences are a valuable part of industrial hemp, as they contain a high amount of several bioactive compounds with potential applications in the agrochemical, cosmetic, pharmaceutical, nutraceutical, and food industries [14,15]. Therefore, the prospect of industrial uses for hemp inflorescences has prompted scientific research to deepen our knowledge of their chemical composition.

Indeed, in the last twenty years, several investigations have been carried out to dissect the metabolic profile of hemp inflorescences [16,17]. From these studies, it emerged that cannabinoids, terpenes, and phenolic compounds represent the most abundant classes of secondary metabolites accumulated in hemp inflorescences [18]. Cannabinoids and terpenes are concentrated in the resin produced by the glandular trichomes, which are mainly located on the surface of female inflorescences [19]. Both these classes of metabolites have been recognized as having therapeutic effects by acting either individually or combined in the so-called “entourage effect”, in which terpenes and cannabinoids are recognized to act synergistically, thus enhancing their beneficial effects [20,21]. In addition to their use as therapeutic agents, terpenes may be used as natural pesticides, fragrances and flavors in cosmetic and cleaning products, and food preservatives [22,23]. The most abundant phenolic compounds in hemp inflorescences are phenolic acids and flavonoids, which are associated with a high number of health-promoting effects mainly related to their antioxidant properties [24]. In *C. sativa*, a synergistic effect between flavonoids and cannabinoids has also been reported [25]. Different factors, such as genotype, climatic conditions, agronomical practices, harvesting stage, and storage conditions, can affect the phytochemical profile of industrial hemp inflorescences. Therefore, understanding the effect of these factors on the accumulation of phytochemical compounds is crucial to obtaining inflorescences enriched with valuable compounds and with high commercial value. To date, several studies have been carried out on the effects of specific agronomical practices, harvesting stages, and storage conditions on the phytochemical composition of hemp inflorescences [26,27,28,29,30], whereas the effects of genotype and cropping season have been poorly investigated. Therefore, the objective of this study was to gain insights for the first time into the effect of genotype, year, and their interaction on the accumulation of secondary metabolites and the antioxidant activity in *C. sativa* inflorescences. To achieve this goal, the inflorescences from six hemp genotypes, including both monoecious and dioecious belonging to chemotypes III and IV, grown in three consecutive cropping years from 2018 to 2020, were characterized through a multi-methodological approach (HPLC, GC-MS, and spectrophotometric assays) for quality and quantity of phenolic compounds, terpenes, cannabinoids, tocopherols, and phytosterols, and for their antioxidant activity. The comparison of different genotypes and the analysis of so many classes of bioactive compounds will provide information that will be helpful in future efforts aimed at maximizing the accumulation of bioactive compounds and favoring the health-promoting uses of hemp inflorescences.

## 2. Results and Discussion

### 2.1. Characterization of the Phytochemical Profile and the Antioxidant Activity in the Inflorescences of the Six Hemp Genotypes Grown in Three Years

The phytochemical profile and the antioxidant activity in the inflorescences of two dioecious genotypes, Carmagnola Selezionata and Fibrante, belonging to chemotype III, three monoecious genotypes, Carmaleonte, Codimono, and Futura 75, belonging to chemotype III, and one monoecious genotype, Santhica 27, belonging to chemotype IV, grown in three consecutive years (2018–2020) were analyzed with respect to total phenolic content (TPC), total flavonoid content (TFC), total antioxidant activity determined spectrophotometrically, and secondary metabolites determined by HPLC and GC/MS. A total of 11 phenolic compounds, including 5 phenolic acids and 6 flavonoids, and 52 non-polar secondary metabolites, including 15 monoterpenes, 23 sesquiterpenes, 2 triterpenes, 5 cannabinoids, 2 tocopherols, and 2 phytosterols, were detected. The total content for each class of secondary metabolites was obtained by summing the individual compounds detected for that class.

#### 2.1.1. Analysis of Variance

As shown in Table 1, two-way ANOVA showed significant effects of genotype (G), year (Y), and genotype by year (G × Y) interaction on all the traits investigated except *trans*-2-pinalol, β-curcumene, α-tocopherol, total tocopherols, γ-sitosterol, and total phytosterols that were affected only by Y, and phytol and total triterpenes that were affected by G and Y but not by G × Y interaction.

**Figure 1:**
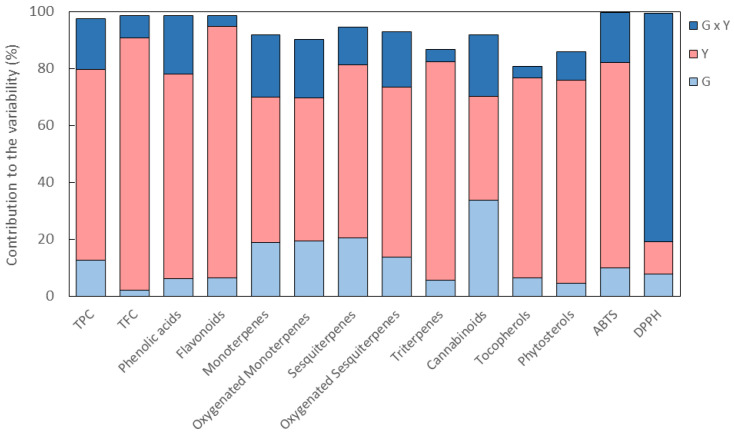
Percentage of contribution to the variability for genotype (G), year (Y), and genotype by year (G × Y) interaction for the classes of metabolites and the antioxidant activity in the inflorescences of the six hemp genotypes grown for three consecutive years (2018–2020). TPC, total phenolic content; TFC, total flavonoid content; ABTS, 2,2-azinobis-(3-ethylbenzothiazoline-6-sulphonic acid) radical scavenging activity; DPPH, 2,2-diphenyl-1-picrylhydrazyl radical scavenging activity.

As shown in Figure 1, the predominant contribution to the variability was from Y for all the classes of metabolites except cannabinoids. In particular, the highest Y contribution, ranging from 67.0% to 88.5%, was observed for the classes of phenolic compounds, determined both spectrophotometrically and by HPLC, and for the classes of triterpenes, tocopherols, and phytosterols (Figure 1 and Table S1). For all these classes of metabolites, G and G × Y interaction accounted for 2.3–12.8% and 3.7–20.5%, respectively, of the variability (Figure 1 and Table S1). As for the other classes of terpenes, the analysis revealed a lower Y contribution, ranging from 50.1% to 61.3%, in favor of an increase in both G and G × Y contributions that ranged from 13.6% to 20.3% and from 13.3% and 21.8%, respectively (Figure 1 and Table S1). For cannabinoids, all the components of variance contributed almost equally to the variability, with G, Y, and G × Y interaction accounting for 34.0%, 36.5%, and 21.4%, respectively, of the variability (Figure 1 and Table S1). The analysis of the individual metabolites revealed that orientin, rutin, and p-hydroxybenzoic acid presented the highest percentage of the Y effect (93.7%, 86.1%, and 82.4%, respectively), whereas the highest contribution to the variability due to the G effect was observed for CBD and CBG (44.6% and 46.2%, respectively) (Table S1). As for the total antioxidant activity, Y gave the highest contribution (71.9%) to the variability of the 2,2-azinobis-(3-ethylbenzothiazoline-6-sulphonic acid) (ABTS) radical scavenging activity, whereas the 2,2-diphenyl-1-picrylhydrazyl (DPPH) radical scavenging activity was mainly affected (80.1%) by the G × Y interaction (Figure 1 and Table S1). To date, there is no other evidence in the literature of the effect of G, Y, and G × Y interaction on the phytochemical profile and the antioxidant activity of hemp inflorescences. However, our findings are in line with data already reported for hemp seeds, for which Y was found to have a major effect on the phenolic and tocopherol content as well as on the antioxidant activity evaluated by the ABTS assay [31,32]. Furthermore, in hemp essential oil, cannabinoids were the only class of phytochemicals mainly affected by G rather than Y, whereas all the other classes of phytochemicals, including monoterpenes and sesquiterpenes, presented significant differences as a function of G, Y, and G × Y interaction [33].

#### 2.1.2. Variability in the Phytochemical Profile Composition among Genotypes

**Figure 2:**
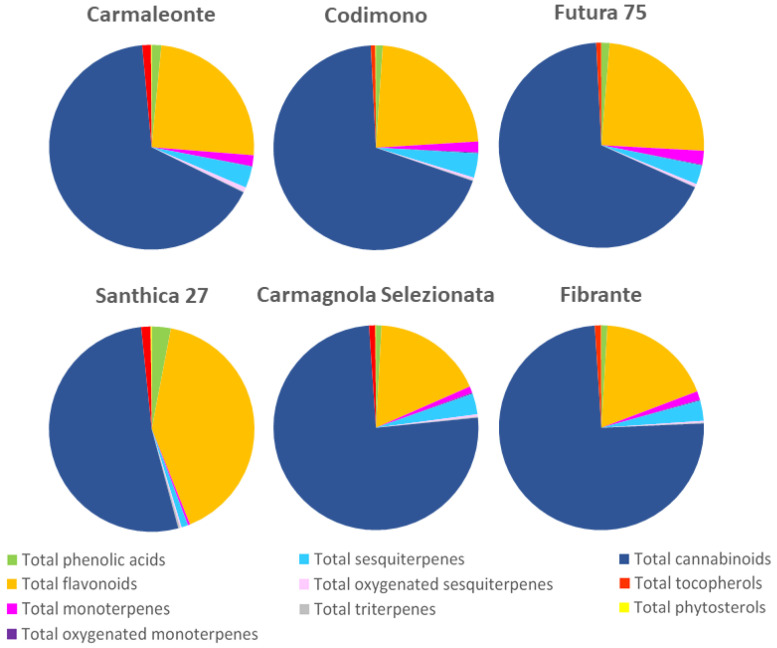
Percentages of the different classes of metabolites detected in the inflorescences of the six hemp genotypes grown for three consecutive years (2018–2020). For each genotype, the percentages were calculated from the means of the three years.

Most of the previous studies carried out on hemp inflorescences have concerned only a few classes of compounds in addition to cannabinoids, mainly phenolic compounds alone [34,35] or together with terpenes [26], or have compared only monoecious [27] or dioecious [29] genotypes. The characterization of different classes of metabolites and the comparison between monoecious and dioecious genotypes carried out in the present study have allowed us to highlight interesting differences in the composition of the phytochemical profile. As shown in Figure 2, cannabinoids were the most represented class of secondary metabolites in all the genotypes investigated. The highest percentages of cannabinoids were detected in the two dioecious Carmagnola Selezionata and Fibrante, in which this class of metabolites accounted for 74.6% and 75.5%, respectively, of the total metabolite content (Figure 2), followed by the monoecious Carmaleonte, Codimono, and Futura 75 with a percentage ranging from 66.3% to 68.9%, whereas the lowest percentage (52.5%) was observed in the monoecious Santhica 27 (Figure 2). Flavonoids were the second most represented class of metabolites and presented an opposite behavior compared to cannabinoids, with the lowest percentages, 17.5% and 18.3%, detected in Carmagnola Selezionata and Fibrante, respectively; intermediate percentages between 23.0% and 24.7% in Carmaleonte, Codimono, and Futura 75; and the highest percentage (40.9%) in Santhica 27 (Figure 2). Compared to the other genotypes, Santhica 27 also presented the highest percentage of phenolic acids (3.0% vs. 0.9–1.5%) and the lowest percentages of sesquiterpenes (1.0% vs. 3.3%) and monoterpenes (0.3% vs. 1.5%) (Figure 2). This peculiar composition of the phytochemical profile, which distinguished Santhica 27 from the other genotypes, could be because this genotype belongs to chemotype IV. Percentages ranging from 0.7% to 1.5% were observed for tocopherols in all six genotypes, whereas oxygenated monoterpenes, oxygenated sesquiterpenes, triterpenes, and phytosterols were the least represented (Figure 2).

#### 2.1.3. Variation in the Phytochemical Content and the Antioxidant Activity Due to Genotype Effect

Complete data concerning TPC, TFC, the individual metabolites detected by HPLC and GC/MS, and the total antioxidant activity are summarized in Table 2. Regarding the differences among genotypes, the lowest TPC and TFC values were detected in Carmaleonte and Santhica 27, whereas no major differences were observed among the other genotypes. Total phenolic acids and total flavonoids detected by HPLC were at their highest levels in Futura 75 and Fibrante, with values 1.2–1.5-fold higher compared to the other genotypes; high levels of total phenolic acids were also detected in Santhica 27. The lowest levels of total phenolic acids were detected in Carmagnola Selezionata, which also presented the lowest levels of total flavonoids together with Carmaleonte and Santhica 27. As for the individual metabolites, phenolic acids were mainly represented by caffeic and vanillic acids, which together accounted for 48.0–66.3% of this class of metabolites, whereas the most abundant flavonoids were orientin and rutin, which represented 63.8–72.3% of the total flavonoids. The analysis of the mean values revealed that the trend observed among genotypes for total phenolic acids and total flavonoids was maintained for almost all the individual metabolites belonging to these classes. Interestingly, TPC and TFC were found to be significantly correlated with the total phenolic compounds (sum of phenolic acids and flavonoids) and the total flavonoids, respectively, determined by HPLC (r = 0.803 *p* ≤ 0.0001 and r = 0.898 *p* ≤ 0.0001, respectively). From these results, it can be assessed that the values obtained from the spectrophotometric assays adequately represent the total phenolic and total flavonoid content in hemp inflorescences, as already observed for other plant and food sources [36,37,38]. Similarly, Izzo and co-authors [35] reported that in hemp inflorescences, TPC significantly correlated with total phenolic compounds determined by spectrometry measurement.

The most abundant classes of terpenes were sesquiterpenes and monoterpenes, which accounted for 51.5–65.7% and 16.9–37.3%, respectively, of total terpenes. The highest levels of total sesquiterpenes were detected in Fibrante, with values that were 1.2–9.7-fold higher than in the other genotypes, whereas Futura 75 presented the highest levels of monoterpenes that were 1.2–16.0-fold higher compared to the other genotypes. Except for triterpenes, Santhica 27 presented the lowest levels of all the classes of terpenes. Overall, a similar trend was observed for the four most abundant individual terpenes, that is, the sesquiterpenes β-caryophyllene and α-humulene and the monoterpenes α-pinene and β-myrcene, which together accounted for 42.4–66.0% of total terpenes. Our results are in line with other studies reporting these four molecules as the most abundant terpenes in hemp inflorescences, although the hemp genotypes analyzed and the amount detected differed from those reported in the present study [39,40,41].

Higher levels of total cannabinoids were detected in the dioecious genotypes compared to the monoecious genotypes. As expected from genotypes belonging to chemotypes III and IV, THC was found to be under the legal limit of 0.3% [9] in all the genotypes analyzed; its highest levels were detected in the dioecious Fibrante and Carmagnola Selezionata, with values 1.4–1.9-fold higher compared to the other chemotype III genotypes and 44.9-fold higher compared to the chemotype IV Santhica 27. In the four genotypes belonging to chemotype III, CBD represented 85.1–89.4% of all the cannabinoids, with the highest level detected in Fibrante (1.6–2.2-fold higher compared to the other genotypes), whereas it accounted only for 28.6% of total cannabinoids in Santhica 27. By contrast, the prevailing cannabinoid in Santhica 27 was CBG, which represented 70.8% of total cannabinoids with levels 3.5–7.1-fold higher compared to chemotype III genotypes. These results are in agreement with the previous definition of chemotypes based on the cannabinoid profile [42].

Tocopherols were mainly represented (77.1–87.6%) by α-tocopherol, and, although not statistically significant due to the high variability among genotypes, 1.5–2.1-fold higher levels of this metabolite were found in Fibrante compared to the other genotypes, whereas the lowest levels were detected in Santhica 27. No major differences were observed among genotypes in the levels of the two phytosterols, campesterol and γ-sitosterol.

Overall, Fibrante presented the highest levels of total phytochemicals, with values 1.4–3.1-fold higher compared to the other genotypes, whereas the lowest levels were detected in Santhica 27.

As for the antioxidant activity, Futura 75 presented the highest ABTS radical scavenging activity and, together with Carmagnola Selezionata, the highest DPPH radical scavenging activity, with values that were 1.1–1.5-fold higher compared to the other genotypes, whereas the lowest values were detected in Carmaleonte and Santhica 27. The antioxidant activity measured by the ABTS assay was found to be higher than that detected by the DPPH assay. This result is not unique. Similar findings were reported by Spano and co-authors in hemp inflorescences harvested at different growth stages [29], as well as in many other antioxidant-rich vegetables, fruits, and beverages [43].

#### 2.1.4. Variation in Phytochemical Content and Antioxidant Activity Due to Year Effect

Regarding the differences among the three cropping years (Table 2), TPC and TFC in 2018 were 1.4- and 2.4-fold higher, respectively, than in 2019 and 2.1-fold higher than in 2020. The highest levels of total phenolic acids were observed in 2019, with values that were 1.5- and 5.0-fold higher compared to 2018 and 2020, respectively. This trend resembled that of vanillic, p-hydroxybenzoic, and caffeic acids, whereas the other phenolic acids were found at their highest levels in 2018, followed by 2019 and 2020. The highest levels of total flavonoids were instead observed in 2018, with values that were 2.4- and 4.6-fold higher compared to 2019 and 2020, respectively. This trend was due to the two most abundant flavonoids, orientin and rutin, and to naringenin, whereas the other flavonoids were more abundant in 2019, followed by 2018 and 2020. Unfortunately, most of the evidence on the effects of the cropping year on the accumulation of phenolic compounds has concerned hemp seeds rather than inflorescences, so it is hard to compare our results with those reported in the literature. However, as for the inflorescences, the seeds of hemp plants grown in different cropping years also showed a variation in the accumulation of phenolic compounds [31,32].

All the classes of terpenes were present at their highest levels in 2018, with values that were 1.9–14.3-fold higher than in 2020 and 4.2–93.0-fold higher than in 2019; this reflected the highest levels detected in 2018 for all the individual terpenes except γ-terpinene. The years 2019 and 2020 differed from each other for the levels of total monoterpenes, total oxygenated monoterpenes, and total sesquiterpenes, which were 25.3-, 44.0-, and 9.9-fold, respectively, higher in the latter compared to the former; this was mainly due to the higher levels of α-pinene, β-myrcene, β-caryophyllene, and α-humulene in 2020 compared to 2019. Similarly, the analysis of the chemical composition of the essential oil extracted by hydrodistillation from eleven hemp genotypes grown for two consecutive years revealed that both monoterpenes and sesquiterpenes presented significant differences as a function of the year of cultivation [33].

Like terpenes, total cannabinoids were also present at their highest levels in 2018, with values 2.2-fold higher compared to 2019 and 2020, which showed no significant difference from each other. The same trend was observed for all the individual cannabinoids except CBG, which in 2019 was found at levels significantly lower compared to 2020. These results are in line with those reported by Spano and co-authors [30], who found a major influence of cropping year on cannabinoid accumulation compared to harvesting stage and fertilization. Furthermore, meaningful differences in the cannabinoid content between two years of cultivation were observed in the essential oil, although these were not statistically significant according to the ANOVA analysis [33].

As for the classes of tocopherols and phytosterols, the levels detected in 2018 were on average 6.5-fold higher compared to the other two cropping years, which instead did not differ from each other except for γ-tocopherol, which was present at higher levels in 2019 compared to 2020.

An overall analysis of the three years revealed that 2018 was characterized by the highest levels of total phytochemicals, with values that were on average 2.4-fold higher than those in 2019 and 2020, which showed no significant difference from each other.

As for the total antioxidant activity, 2020 presented the lowest values regardless of the assay used, whereas the highest ABTS radical scavenging activity was observed in 2019, with values 1.1- and 3.0-fold higher compared to 2018 and 2020, respectively, and the highest DPPH radical scavenging activity was observed in 2018, with values 1.1- and 1.2-fold higher compared to 2019 and 2020, respectively. Differences in the antioxidant activity in the hemp inflorescences due to the cropping year have also been reported by Spano and co-authors [30], however, only for the DPPH assay, whereas no differences were observed for the ABTS assay. The different responses of the DPPH and ABTS assays to the cropping year observed by the authors and in the present study between 2018 and 2019 may be ascribable to the ability of the two assays to detect the antioxidant activity of different molecules (see Section 2.2).

In the present study, the main difference in environmental conditions between 2018 and the other two cropping years was the regularity of rainfall and temperatures throughout the plant growth cycle (see Section 3). These weather conditions might be responsible for the high levels of almost all the classes of phytochemicals in the inflorescences of hemp plants grown in 2018. As for the differences between 2019 and 2020, a very low rainfall characterized the month of September 2020, which may have caused a lower accumulation of phenolic compounds compared to 2019. In this regard, Irakli and co-authors [31] investigated the phytochemical composition of seeds from seven hemp cultivars grown for three consecutive years and found that the highest values of phenolic compounds occurred in the cropping year with the highest rainfall during the late period of inflorescence development. Accordingly, Menga and co-authors [32] in a two-year study highlighted a positive correlation between rainfall and phenolic accumulation in the seeds of three different hemp genotypes. By contrast, the high rainfall that characterized 2019 may be responsible for the lower accumulation of terpenes, which is known to be promoted by low rainfall and deficit irrigation [44,45]. Less clear is the effect of environmental conditions on the accumulation of cannabinoids. In this regard, the effect of water availability on cannabinoid content is still debated. Caplan and co-authors [46] reported that drought stress increased the accumulation of major cannabinoids, whereas Spano and co-authors [30] observed an increase in THC and CBD when continuous irrigation was applied; furthermore, other authors did not observe a clear response of cannabinoid content in relation to water availability [47].

However, further investigations are needed to dissect the role of the different meteorological parameters on the accumulation of the different classes of phytochemicals in hemp inflorescences.

### 2.2. Correlation between Phytochemicals and Antioxidant Activity

**Figure 3:**
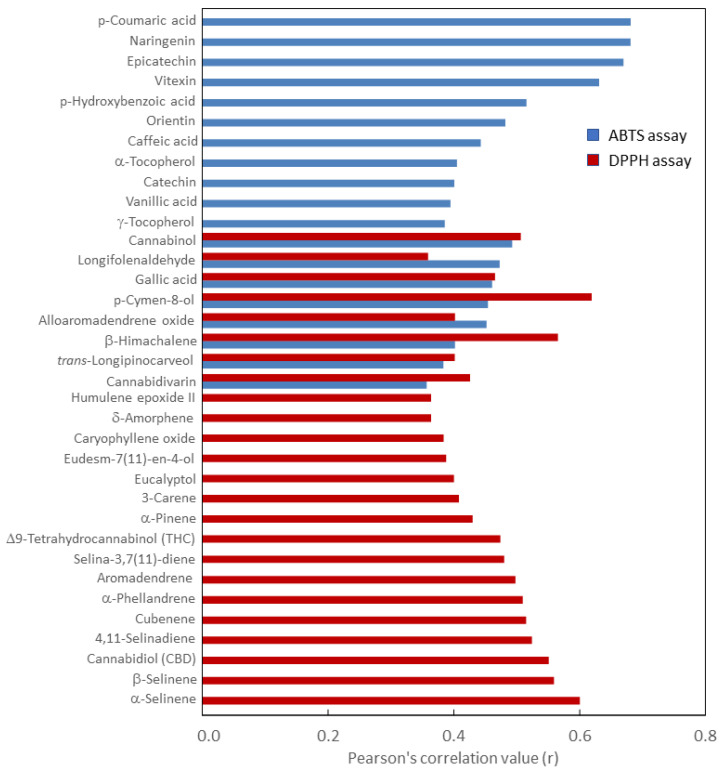
Pearson’s correlation value (r) between each metabolite and the antioxidant activity measured by both the ABTS and the DPPH assays. All the correlations are statistically significant at *p* ≤ 0.01.

A pairwise correlation analysis was carried out to investigate the relationship between the metabolites detected and antioxidant activity. As shown in Figure 3, 19 and 24 metabolites significantly (*p* ≤ 0.01) correlated with the ABTS and DPPH radical scavenging activities, respectively. Interestingly, only eight metabolites, which included gallic acid, five oxygenated terpenes (p-cymen-8-ol, *trans*-longipinocarveol, β-himachalene, alloaromadendrene, and longifolenaldehyde), and two cannabinoids (cannabidivarin and cannabinol), showed a significant correlation with both assays (r = 0.36–0.62) (Figure 3). Conversely, metabolites belonging to the classes of phenolic compounds and tocopherols mainly correlated with the ABTS assay (r = 0.39–0.68), whereas the DPPH assay mostly correlated with terpenes, THC, and CBD (r = 0.36–0.60) (Figure 3). Overall, these findings explained the antioxidant activity detected in the six genotypes and the three cropping years (Table 2). Indeed, genotypes and years that presented high levels of phenolic compounds also presented high ABTS radical scavenging activity, while high DPPH antioxidant activity was detected in those genotypes and years with high levels of terpenes and cannabinoids (Table 2).

This is the first time that a clear and detailed picture emerges of the contribution of different classes of phytochemicals to the antioxidant activity in hemp inflorescences. Unfortunately, limited information is available on the antioxidant activities of lipophilic compounds from hemp inflorescences since most of the studies have been limited to the evaluation of a correlation between the antioxidant activity and the phenolic compounds [26,29]. However, based on their findings, Spano and co-authors [29] hypothesized that cannabinoids could also be involved in the antioxidant activity of hemp inflorescences, as they observed that high antioxidant activity was detected at those growth stages at which levels of polyphenols and cannabinoids in the inflorescences were also high. These and our findings agree with evidence already reported in the literature that, like phenolic compounds, cannabinoids and terpenes may exert their beneficial effects on human health through their antioxidant properties [48,49]. The phytochemical profile of hemp inflorescences is complex, and the ABTS assay proved to be more sensitive and more suitable for the detection of water-soluble antioxidants compared to the DPPH assay [50]. Consistently, in hemp seeds, the ABTS assay was found to be highly correlated with TPC and some phenolic compounds, whereas a lower correlation was found with γ-tocopherol [31], similar to what was observed in the present study (Figure 3). Therefore, an integrated use of different antioxidant assays is needed to provide a complete picture of the antioxidant potential of both lipophilic and hydrophilic phytochemicals in hemp inflorescences.

### 2.3. Multivariate Analysis

The principal component analysis (PCA) revealed that the first three principal components (PCs) represented more than 79% of the variability in the dataset, with PC1 and PC2 explaining 59.0% and 12.5%, respectively, of the total variability (Table S2). Except for a few phenolic acids, flavonoids, and antioxidant activity, all the metabolites were positively correlated with the first component (PC1) (Table S2). ABTS radical scavenging activity, p-hydroxybenzoic acid, p-coumaric acid, epicatechin, vitexin, and naringenin were the most important traits positively correlated with PC2 (Table S2).

**Figure 4:**
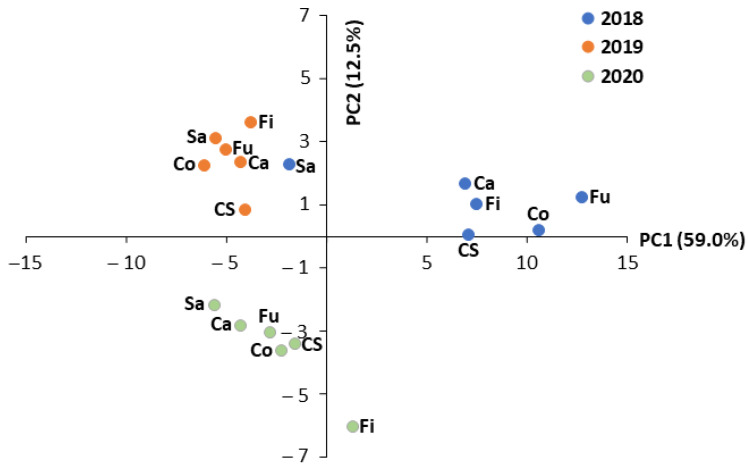
Principal component analysis (PCA) of the different traits analyzed in the inflorescences of the six hemp genotypes grown for three consecutive years (2018–2020). PC1, first principal component; PC2, second principal component; Ca, Carmaleonte; Co, Codimono; CS, Carmagnola Selezionata; Fi, Fibrante; Fu, Futura 75; Sa, Santhica 27.

With few exceptions, the score plot of the PCA highlighted a grouping of samples based on the cropping year (Figure 4). The difference between 2018 and the other two years was particularly evident in the PC1 direction, with all the genotypes grown in 2018 clustered on the positive side of the PC1 axis, except for Santhica 27, which was positioned on the negative side together with the genotypes grown in 2019 (Figure 4). These latter genotypes and those grown in 2020 were well differentiated on the PC2, respectively on the positive and negative sides of the PC2 axis (Figure 4). Fibrante grown in 2020 was separated from the other genotypes grown in the same year on both the PC1 and PC2 axes (Figure 4).

**Figure 5:**
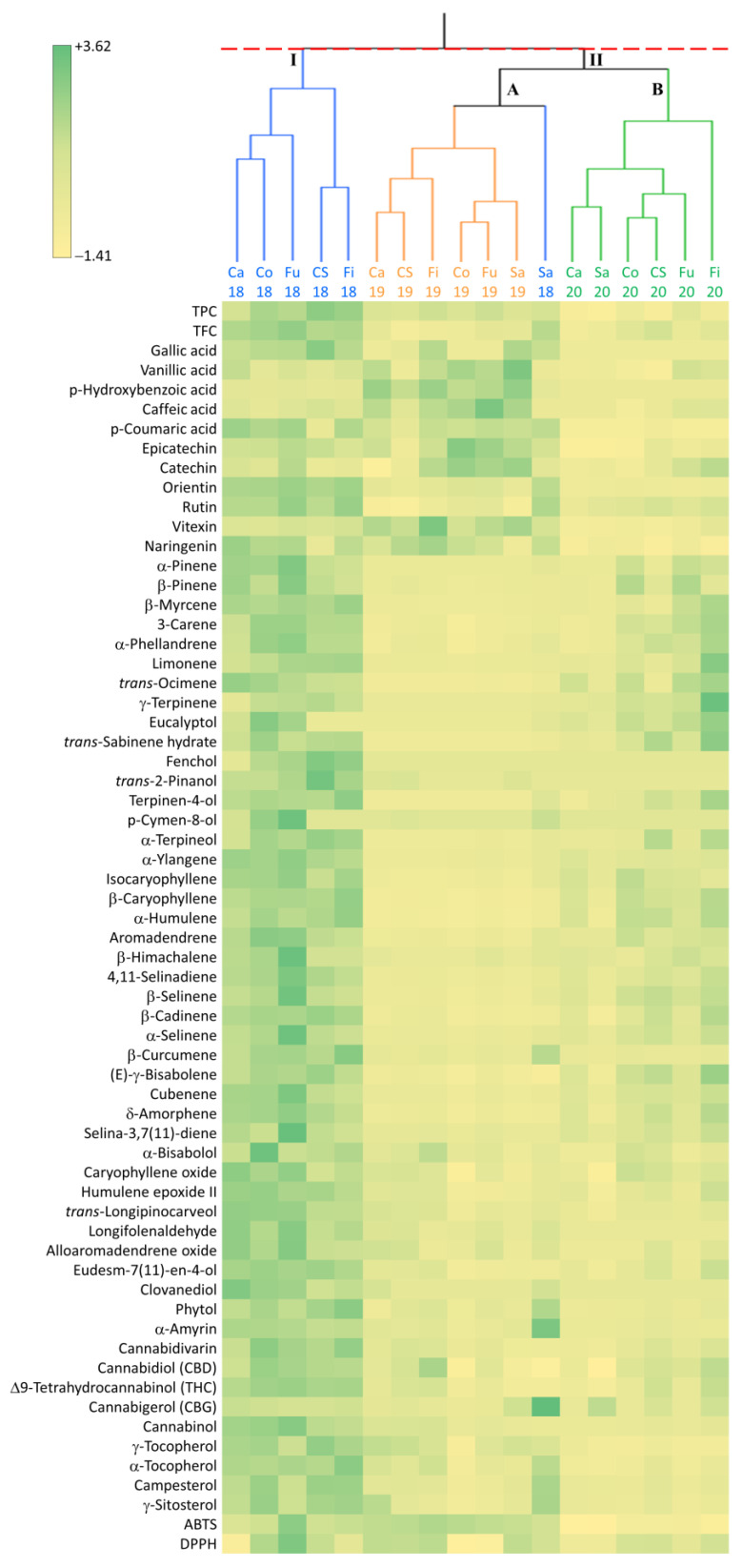
Hierarchical cluster analysis (HCA) dendrogram (Ward’s method) and pattern map for the different traits analyzed in the inflorescences of the six hemp genotypes grown for three consecutive years (2018–2020). HCA was carried out using the first seven principal components (PC) having an eigenvalue ≥ 1. Ca, Carmaleonte; Co, Codimono; CS, Carmagnola Selezionata; Fi, Fibrante; Fu, Futura 75; Sa, Santhica 27.

As shown in Figure 5, the hierarchical cluster analysis (HCA) confirmed the grouping of samples observed through the PCA. In particular, the HCA identified two clusters, I and II; except for Santhica 27, cluster I consisted of all the genotypes grown in 2018 that were characterized by high to very high values of all the traits investigated except for a few metabolites, such as vanillic, p-hydroxybenzoic and caffeic acid, vitexin, and CBG, that were found at low levels (Figure 5). Cluster II comprised two subclusters, A and B. The subcluster A included all the genotypes grown in 2019 that were characterized by high levels of most phenolic compounds and antioxidant activity and low levels of all the other metabolites, and Santhica 27 grown in 2018 that had a profile similar to genotypes grown in 2019 except for the high levels observed also for β-curcumene, phytol, α-tocopherol, and phytosterols and the very high levels of α-amyrin and CBG (Figure 5). Subcluster B included all the genotypes grown in 2020 and was characterized by low levels of phenolic compounds and antioxidant activity and low to intermediate levels of all the other metabolites investigated (Figure 5). In this subcluster, Fibrante differed from the other genotypes for the high levels of most monoterpenes, oxygenated monoterpenes, sesquiterpenes, and CBD (Figure 5).

### 2.4. Phytochemical Profile Stability

**Figure 6:**
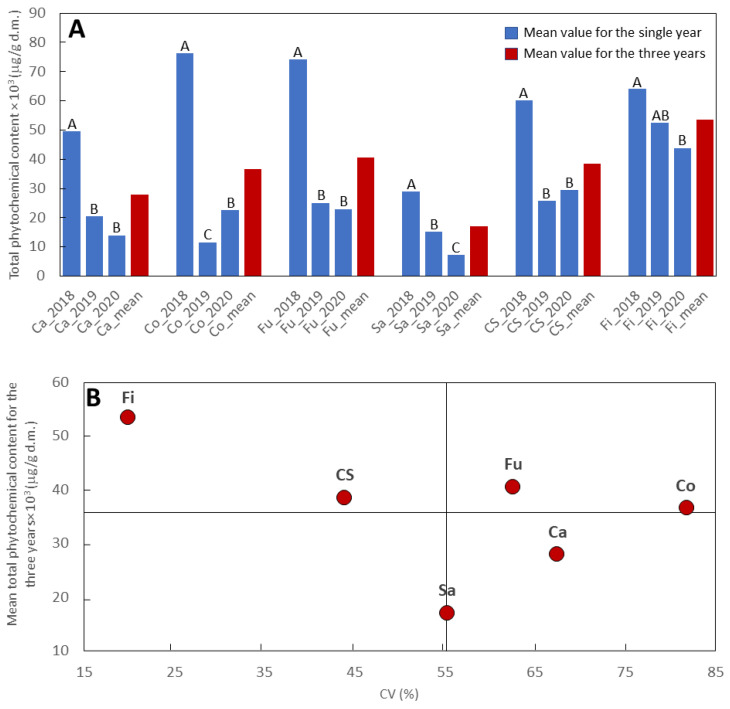
Phytochemical profile stability in the six hemp genotypes over the three consecutive years (2018–2020). (**A**) Mean values for each year and for the three years. For each genotype, different capital letters represent significant differences among years (Tukey’s test, *p* < 0.05). (**B**) Mean values for the three years plotted against the coefficient of variation (CV); Ca, Carmaleonte; Co, Codimono; CS, Carmagnola Selezionata; Fi, Fibrante; Fu, Futura 75; Sa, Santhica 27.

The great and significant Y effect gave reason to evaluate the performance of each genotype over the three cropping years. For this purpose, the total phytochemical content for each year and the mean of three years were reported in Figure 6A. In addition, for each genotype, the mean total phytochemical content was plotted against the variation coefficient over three years (Figure 6B). The results reported in Figure 6 showed that, with regard to the total phytochemical accumulation, the dioecious genotypes presented a more constant performance over the three years than the monoecious genotypes, with the highest and most stable phytochemical content observed in Fibrante. The strong difference between the performance of 2018 and that of the other two years that characterized the three monoecious genotypes, Carmaleonte, Codimono, and Futura 75, was responsible for the low-performance stability of these genotypes over the three years, whereas the monoecious Santhica 27 presented better stability compared to the other monoecious genotypes, but the total phytochemical accumulation over the three cropping years was the lowest among all genotypes investigated (Figure 6).

## 3. Materials and Methods

### 3.1. Plant Material and Growing Conditions

**Figure 7:**
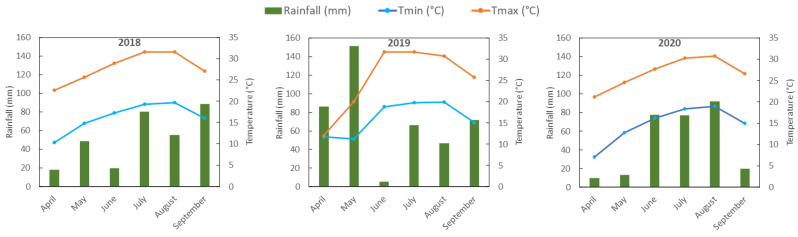
Weather parameters: rainfall (mm), minimum temperature (Tmin, °C), and maximum temperature (Tmax, °C) during the three cropping seasons.

In the present study, six industrial hemp genotypes, four monoecious, Codimono, Carmaleonte, Futura 75, and Santhica 27, and two dioecious, Fibrante and Carmagnola Selezionata, were grown at the experimental field of the Research Centre for Cereal and Industrial Crop of Rovigo, Rovigo, Italy (45.089092, 11.767419) for three consecutive years from 2018 to 2020. The six genotypes were sown on 31 May 2018, 19 April 2019, and 23 April 2020, following a complete randomized block design with three parcel replicates. Briefly, the sowing density was 40 kg/ha with an inter-row spacing of 25 cm on a large plot (20 m^2^) fertilized in pre-sowing according to common agronomical practice. Manual weed control was used during the initial stages of crop development. Figure 7 reports the monthly rainfall and the minimum and maximum temperatures from April to September for each cropping season. The total rainfall registered during the cropping seasons of 2018, 2019, and 2020 was 309, 427, and 288 mm, respectively (Figure 7). The air temperature generally increased from April to August, with an average minimum temperature of 16.2 °C, 16.1 °C, and 14.7 °C in 2018, 2019, and 2020, respectively, whereas the average maximum temperature was 27.8 °C, 25.3 °C, and 26.8 °C, respectively (Figure 7).

The inflorescences of the monoecious and female dioecious plants were harvested at the phenological stage corresponding to seed maturity (50% of seeds were hard), encoded as BBCH 67 [51]. For each replicate, five inflorescences were randomly collected. Immediately after being cut from the plant, each inflorescence was immersed in liquid nitrogen. Each sample was lyophilized and stored for further analysis. Before the analysis, the lyophilized samples were sieved and ground into powder using a planetary mill with jar balls (Pulverisette 7, Fritsch, Milan, Italy).

### 3.2. Analysis of Phenolic Compounds

Phenolic compounds were extracted essentially as reported in [32]. Briefly, 100 mg of the ground sample were dissolved in 5 mL of a methanol:water (80:20 *v*/*v*) solution acidified with 1% HCl; the suspension was vortexed and ultrasonicated for 30 min at room temperature. After centrifugation at 1000× *g* for 15 min at room temperature, the supernatant was collected into clean tubes and stored at −20 °C until further analysis.

#### 3.2.1. Spectrophotometric Determination of TPC and TFC

TPC was determined using the Folin-Ciocalteu assay according to [32]. Briefly, 200 µL extract were added to 1.5 mL of 10-fold diluted Folin-Ciocalteu reagent, then the solution was equilibrated for 5 min and mixed with 1.5 mL of 6% (*w*/*v*) sodium carbonate solution. After incubation at room temperature for 90 min, the absorbance of the solution at 725 nm was measured. TPC was expressed as mg of ferulic acid equivalents/g of dry matter (d.m.).

TFC was determined according to [52] with minor modifications. Briefly, 250 µL of each extract were added to 1.25 mL of distilled water and 75 µL of 5% (*w*/*v*) NaNO_2_. Six minutes later, 150 µL of 10% AlCl_3_ (*w*/*v*) were added to the mixture. Then, 500 µL of 1 M NaOH were added after five minutes, and the total volume was made up to 3 mL with distilled water. The solution was mixed, and its absorbance at 510 nm was measured. All assays were performed in triplicate, and the results were reported as mg of catechin equivalents/g d.m.

#### 3.2.2. Determination of Phenolic Acids and Flavonoids by HPLC

Phenolic acids and flavonoids were separated, identified, and quantified according to [53] by using an HPLC system (Series 1200; Agilent Technologies, Waldbronn, Germany) equipped with a diode array detector (Agilent Technologies, Waldbronn, Germany) with few modifications. For the separation, a C18 column (Zorbax SB-C18; 100 mm × 2.1 mm × 1.8 µm; Agilent, Santa Clara, CA, USA) was used. The temperature of the column oven was set to 35 °C. A gradient elution was used with a mobile phase of acetonitrile (solution A) and 1% acetic acid (solution B), as follows: 0 min = 100% B; 12 min = 85% B; 20 min = 50% B; 22 min = 0% B; 24 min = 100% B; isocratic elution of B 24–30 min. The flow rate of the mobile phase was 0.4 mL/min, and the injection volume was 1 µL. Peaks were detected at 280 nm and 320 nm, and metabolites were identified through their characteristic UV/Vis spectra and comparison of the retention times with those of authentic standards. The quantification of each phenolic acid and flavonoid was calculated using the corresponding calibration curve. All assays were performed in triplicate, and the results were reported as μg/g d.m.

### 3.3. Analysis of Non-Polar Secondary Metabolites by GC/MS

Non-polar secondary metabolites were extracted and determined according to [54], with minor modifications. The extraction was carried out using the accelerated solvent extraction Dionex ASE350 (Thermo Fisher Scientific Inc., Waltham, MA, USA). Two hundred and fifty milligrams of each ground sample were weighed in a stainless steel cell, added to 10 μL of internal standard (pentadecane 11.5 mg/mL), and extracted with *n*-hexane using the following program conditions: 1 cycle with 5 min of heating and 15 min of static extraction at a temperature of 50 °C and a pressure of about 10 MPa. Two microliters of extract were injected into the GC/MS system (Agilent 6890A coupled to a Triple Quadrupole Mass Spectrometer 7000B, Agilent Technologies, Santa Clara, CA, USA). For the separation, an HP-5ms capillary column (60 m × 0.25 mm i.d. × 0.25 μm film thickness) was used. The injection and transfer line temperatures were set at 280 °C, whereas the source temperature was set at 240 °C. The oven temperature programming began at 60 °C for 1 min, followed by a 5 °C/min increase up to 220 °C maintained for 1 min and an 11 °C/min increase up to 280 °C maintained for 20 min. Helium was used as a carrier gas at a constant flow rate of 1 mL/min. The MS operated in EI at 70 eV with a mass range of 50–700 amu. As reported in Table S3, metabolites were identified by comparison of their mass spectra with those of NIST11 and, whenever possible, by comparison with those of the authentic standard available, and confirmed by comparison of their experimental retention indices (determined using homologous series of C8–C34 alkanes) with those reported in the literature [55,56]. Semi-quantification was made by peak normalization with those of the internal standard added and the sample weight. All assays were performed in triplicate, and the results were expressed as μg/g d.m.

### 3.4. Determination of the Total Antioxidant Activity

The antioxidant activity was determined according to the ABTS and DPPH radical scavenging assays, essentially as described in Iannucci et al. [57]. Ten milligrams of ground sample were dissolved in 1 mL of ethanol:water (50:50 *v*/*v*) solution, mixed for 30 min at room temperature, and centrifuged at 2900× *g* at 4 °C for 10 min.

#### 3.4.1. ABTS Radical Scavenging Assay

For the ABTS assay, the ABTS radical cation (ABTS^•+^) was prepared daily by the reaction of the ABTS solution (7 mM in water) with potassium persulfate (2.45 mM final concentration in water). After incubation for 16 h at room temperature in the dark, the ABTS^•+^ solution was diluted with ethanol to obtain an absorbance value of 0.8 at 734 nm. For the determination of the ABTS^•+^ scavenging activity, 20 µL of each sample were mixed with 4.98 mL of diluted ABTS^•+^ solution. The reaction mixture was incubated at 30 °C for 20 min in the dark, and then the absorbance at 734 nm was measured.

#### 3.4.2. DPPH Radical Scavenging Assay

For the DPPH assay, a DPPH radical (DPPH^•^) solution having an absorbance value of 0.80 at 525 nm was prepared daily by dissolving 5 mg of DPPH in 100 mL of a methanol/water mixture (50:50, *v*/*v*). For the determination of the DPPH^•^ scavenging activity, 20 μL of each sample were added to 4.98 mL of DPPH^•^ solution. The reaction mixture was incubated at 30 °C in the dark for 30 min, and then the absorbance at 517 nm was measured.

For both assays, 6-hydroxy-2,5,7,8-tetramethyl-chroman-carboxylic acid (Trolox), a water-soluble analogue of vitamin E, was used as the reference standard. All assays were performed in triplicate, and the results were expressed as mg of Trolox equivalent/g d.m.

### 3.5. Statistical Analysis

A two-way analysis of variance (ANOVA) was carried out to determine the effect of genotype (G), cropping year (Y), G × Y interaction, and residual variance (ε) on the traits investigated. Significant differences among the means were evaluated using the Tukey’s multiple test (*p* ≤ 0.05). Relationships between individual variables were examined using Pearson’s correlation coefficients (*p* ≤ 0.05), while pairwise correlation between metabolites and antioxidant activity was determined at *p* ≤ 0.01. The traits were subjected to the PCA based on correlations, and only the eight components with an eigenvalue ≥ 1 were selected [58]. The eigenvectors of these principal components were used for the HCA. Entries were clustered using Ward’s minimum-variance method, and a color map based on the standardized mean values of the metabolites was constructed to identify the clusters. The reference line on the dendrogram for the identification of the group to which each entry belonged was derived by the automatic truncation available as part of the software used. All the statistical analyses were performed using the JMP software, version 8.0 (SAS Institute Inc., Cary, NC, USA).

## 4. Conclusions

The present study represents the first attempt at characterizing the effects of genotype, cropping year, and their interaction on the phytochemical content and antioxidant activity of hemp inflorescences, thus contributing to a broader understanding of the plasticity and stability of these biochemical traits across different genotypes. Overall, our findings highlighted a strong year effect but also showed that the phytochemical accumulation in Fibrante remained rather stable over the three years, resulting in the best profile among all the genotypes analyzed. In particular, its profile was characterized by the highest levels of CBD, α-humulene, and β-caryophyllene, which may confer on the inflorescences of this genotype a great economic value due to the important pharmacological properties of these metabolites. Indeed, CBD is a non-psychoactive cannabinoid that exhibits wide therapeutic properties, including neuroprotective, cardioprotective, anti-inflammatory, analgesic, anxiolytic, and anticancer effects [59]. Similarly, β-caryophyllene and α-humulene have been shown to possess anti-inflammatory properties [60], whereas β-caryophyllene also exhibits anxiolytic and anti-depressant activities thanks to its ability to bind to the cannabinoid receptor subtype 2 (CB2) [61], thus suggesting an additive or synergistic effect with CBD in alleviating anxiety and depressive disorders. In addition, Santhica 27, despite its worst phytochemical profile over the three years, presented a high level of CBG, known for its antibacterial, antifungal, and anti-inflammatory properties [62]. Therefore, these genotypes represent excellent candidates for use in future breeding programs aimed at the selection of new hemp genotypes with improved levels of these phytochemicals. Future studies will be useful to further explore the biological activity and safety of these molecules of interest and evaluate the potential applications of the improved hemp genotypes for the development of food and beverages with health benefits in the flavoring sector as well as in the cosmetics and pharmaceutical industries. Our findings and future findings will promote the circular economy of the hemp industry by converting hemp waste into new products with high added value.

## Figures and Tables

**Table 1:** Analysis of variance for total phenolic content (TPC), total flavonoid content (TFC), and the 2,2-azinobis-(3-ethylbenzothiazoline-6-sulphonic acid) (ABTS) and 2,2-diphenyl-1-picrylhydrazyl (DPPH) radical scavenging activities determined spectrophotometrically, and for each class of secondary metabolites and each individual metabolite determined by HPLC and GC/MS in the six hemp genotypes grown in three consecutive years (2018–2020).

Trait	Genotype (G) (*df* 5)	Year (Y) (*df* 2)	G × Y (*df* 17)
TPC	***	***	***
TFC	***	***	***
***Phenolic acids***			
Gallic acid	***	***	***
Vanillic acid	***	***	***
p-Hydroxybenzoic acid	***	***	***
Caffeic acid	***	***	***
p-Coumaric acid	***	***	***
Total phenolic acids	***	***	***
***Flavonoids***			
Epicatechin	***	***	***
Catechin	***	***	***
Orientin	***	***	***
Rutin	***	***	***
Vitexin	***	***	***
Naringenin	***	***	***
Total flavonoids	***	***	***
***Monoterpenes***			
α-Pinene	***	***	***
β-Pinene	***	***	***
β-Myrcene	***	***	**
3-Carene	***	***	**
α-Phellandrene	***	***	***
Limonene	***	***	***
*trans*-Ocimene	***	***	***
γ-Terpinene	***	***	***
Total monoterpenes	***	***	***
***Oxygenated monoterpenes***			
Eucalyptol	***	***	***
*trans*-Sabinene hydrate	***	***	***
Fenchol	***	***	***
*trans*-2-Pinanol	NS	***	NS
Terpinen-4-ol	***	***	***
p-Cymen-8-ol	***	***	***
α-Terpineol	***	***	***
Total oxygenated monoterpenes	***	***	***
***Sesquiterpenes***			
α-Ylangene	***	***	***
Isocaryophyllene	***	***	**
β-Caryophyllene	***	***	***
α-Humulene	***	***	***
Aromadendrene	***	***	***
β-Himachalene	***	***	***
4,11-Selinadiene	***	***	***
β-Selinene	***	***	***
β-Cadinene	***	***	***
α-Selinene	***	***	***
β-Curcumene	NS	***	NS
(*E*)-γ-Bisabolene	***	***	***
Cubenene	***	***	***
δ-Amorphene	*	***	*
Selina-3,7(11)-diene	*	***	**
Total sesquiterpenes	***	***	***
***Oxygenated sesquiterpenes***			
α-Bisabolol	***	***	***
Caryophyllene oxide	***	***	***
Humulene epoxide II	***	***	***
*trans*-Longipinocarveol	***	***	***
Longifolenaldehyde	***	***	***
Alloaromadendrene oxide	***	***	***
Eudesm-7(11)-en-4-ol	***	***	***
Clovanediol	***	***	***
Total oxygenated sesquiterpenes	***	***	***
***Triterpenes***			
Phytol	**	***	NS
α-Amyrin	***	***	***
Total triterpenes	*	***	NS
***Cannabinoids***			
Cannabidivarin	***	***	***
Cannabidiol (CBD)	***	***	***
Δ9-Tetrahydrocannabinol (THC)	***	***	***
Cannabigerol (CBG)	***	***	***
Cannabinol	***	***	***
Total cannabinoids	***	***	***
***Tocopherols***			
γ-Tocopherol	***	***	***
α-Tocopherol	NS	***	NS
Total tocopherols	NS	***	NS
***Phytosterols***			
Campesterol	**	***	*
γ-Sitosterol	NS	***	NS
Total phytosterols	NS	***	NS
Total phytochemicals	***	***	***
ABTS	***	***	***
DPPH	***	***	***

*** *p* ≤ 0.001; ** *p* ≤ 0.01; * *p* ≤ 0.05; NS, not significant.

**Table 2:** Mean values for the total phenolic content (TPC), total flavonoid content (TFC), and the 2,2-azinobis-(3-ethylbenzothiazoline-6-sulphonic acid) (ABTS) and 2,2-diphenyl-1-picrylhydrazyl (DPPH) radical scavenging activities determined spectrophotometrically, and for the content of each individual metabolite and each class of secondary metabolites determined by HPLC and GC/MS in the six hemp genotypes grown in three consecutive years (2018–2020).

Trait ^1^	GENOTYPE						YEAR		
	Carmaleonte	Codimono	Futura 75	Santhica 27	Carmagnola Selezionata	Fibrante	2018	2019	2020
TPC	22.9 b	28.2 a	29.8 a	21.9 b	30.2 a	30.8 a	37.7 A	26.3 B	17.9 C
TFC	8.9 c	9.9 ab	10.7 a	8.7 c	9.5 bc	9.4 bc	15.2 A	6.2 C	7.1 B
***Phenolic acids***									
Gallic acid	44.1 b	60.2 b	64.2 b	118.4 a	126.0 a	126.3 a	196.2 A	73.3 B	0.0 C
Vanillic acid	124.3 a	113.6 a	130.1 a	138.6 a	67.2 b	119.1 a	101.1 B	169.9 A	75.5 C
p-Hydroxybenzoic acid	76.6 a	40.6 b	53.1 ab	80.9 a	35.3 b	73.6 a	16.4 B	163.6 A	0.0 B
Caffeic acid	127.9 b	127.2 b	217.2 a	131.6 b	95.5 c	139.4 b	93.5 B	257.4 A	68.4 C
p-Coumaric acid	55.1 b	48.2 b	59.4 a	44.0 b	15.3 c	51.8 b	84.5 A	51.4 B	1.1 C
Total phenolic acids	428.1 b	389.8 bc	524.0 a	513.5 a	339.3 c	510.2 a	491.7 B	715.7 A	145.0 C
***Flavonoids***									
Epicatechin	666.9 b	1106.8 a	1139.6 a	747.0 b	639.8 b	695.1 b	953.4 B	1246.2 A	298.0 C
Catechin	458.1 c	711.7 b	871.2 a	707.4 b	475.9 c	758.3 ab	599.1 B	832.5 A	559.7 B
Orientin	2880.3 bc	3386.6 ab	3741.4 a	2215.7 d	2710.2 cd	3485.7 ab	7413.3 A	1420.4 B	376.2 C
Rutin	1939.9 c	2516.6 b	3227.7 a	2255.3 bc	2177.6 bc	3176.6 a	5019.4 A	791.8 C	1835.6 B
Vitexin	845.1 bc	560.9 c	856.9 bc	1001.9 b	661.0 c	1504.3 a	753.6 B	1786.9 A	0.175 C
Naringenin	143.8 a	141.9 a	135.8 a	81.0 b	92.7 b	160.2 a	202.7 A	156.4 B	0.019 C
Total flavonoids	6934.1 c	8424.6 b	9972.8 a	7008.2 c	6757.2 c	9780.3 a	14,941.6 A	6234.4 B	3262.6 C
***Monoterpenes***									
α-Pinene	217.9 bc	295.2 ab	414.9 a	17.5 d	106.4 cd	149.4 bcd	463.1 A	2.2 C	135.4 B
β-Pinene	59.3 b	68.9 b	113.6 a	5.6 c	43.7 bc	29.0 bc	102.0 A	6.9 C	51.2 B
β-Myrcene	129.2 b	122.1 bc	192.6 ab	8.3 c	110.0 bc	257.6 a	300.0 A	2.9 C	107.0 B
3-Carene	5.1 bc	11.2 ab	13.2 a	3.1 c	9.3 abc	13.8 a	16.1 A	2.2 C	9.5 B
α-Phellandrene	3.9 bc	9.5 ab	11.8 a	3.1 c	9.5 ab	11.9 a	14.9 A	2.1 C	7.9 B
Limonene	17.8 bc	31.6 bc	47.9 b	5.9 c	41.0 b	94.2 a	74.2 A	3.4 C	41.5 B
*trans*-Ocimene	36.3 a	35.4 a	33.1 a	4.0 b	12.8 b	33.0 a	44.7 A	0.2 C	32.4 B
γ-Terpinene	8.3 c	54.6 b	63.0 b	7.9 c	63.7 b	143.8 a	75.4 B	0.1 C	95.2 A
Total monoterpenes	477.8 bc	628.6 abc	890.2 a	55.5 d	396.4 c	732.7 ab	1090.5 A	19.9 C	480.2 B
***Oxygenated monoterpenes***									
Eucalyptol	4.8 c	17.0 a	13.9 ab	1.7 c	2.4 c	10.7 b	12.6 A	0.0 B	12.6 A
*trans*-Sabinene hydrate	5.9 cd	13.9 abc	8.3 bcd	1.2 d	16.1 ab	21.3 a	18.4 A	0.0 B	15.0 A
Fenchol	0.0 b	2.5 ab	3.5 ab	0.0 b	5.7 a	4.7 a	8.2 A	0.0 B	0.0 B
*trans*-2-Pinanol	3.3 a	2.6 a	4.4 a	0.7 b	10.0 a	5.0 a	11.9 A	1.1 B	0.0 B
Terpinen-4-ol	3.6 bc	5.6 bc	5.5 bc	1.6 c	6.3 b	12.1 a	12.3 A	0.0 C	5.0 B
p-Cymen-8-ol	0.3 b	2.0 ab	3.1 a	0.6 b	0.1 b	0.0 b	3.0 A	0.1 B	0.0 B
α-Terpineol	5.4 cd	13.6 abc	11.7 bcd	1.8 d	23.9 a	21.8 ab	27.5 A	0.2 C	11.3 B
Total oxygenated monoterpenes	23.2 bc	57.2 ab	50.3 ab	7.7 c	64.5 a	75.6 a	93.8 A	1.4 C	43.9 B
***Sesquiterpenes***									
α-Ylangene	7.9 a	7.0 a	8.7 a	0.7 b	6.2 a	5.6 a	15.0 A	0.2 C	2.9 B
Isocaryophyllene	9.8 a	12.0 a	11.4 a	1.7 b	6.1 ab	8.4 a	17.4 A	0.4 C	6.8 B
β-Caryophyllene	315.8 c	455.7 b	384.8 bc	53.8 d	383.0 bc	608.1 a	695.8 A	21.4 C	383.4 B
α-Humulene	388.7 c	642.3 ab	431.1 bc	59.4 d	596.5 abc	784.6 a	871.3 A	41.7 C	538.2 B
Aromadendrene	13.0 b	27.5 a	24.1 a	4.8 c	13.1 b	13.9 b	34.2 A	1.8 C	12.2 B
β-Himachalene	5.7 bc	6.0 b	15.6 a	1.3 c	3.5 bc	3.6 bc	13.8 A	0.6 C	3.4 B
4,11-Selinadiene	15.2 b	17.2 b	27.7 a	2.5 c	19.3 ab	18.5 ab	36.6 A	1.7 C	11.9 B
β-Selinene	18.2 bc	25.1 b	42.3 a	5.4 c	25.2 b	22.7 b	42.0 A	3.5 C	24.0 B
β-Cadinene	8.0 b	8.7 ab	8.6 ab	0.9 c	12.7 a	13.0 a	16.9 A	1.7 C	7.3 B
α-Selinene	19.5 bc	27.6 b	48.3 a	4.9 c	27.8 b	24.6 b	53.4 A	3.0 C	20.0 B
β-Curcumene	19.8 a	31.8 a	33.6 a	23.2 a	25.1 a	48.4 a	81.8 A	5.3 B	3.9 B
(*E*)-γ-Bisabolene	10.2 b	14.1 ab	12.6 ab	1.2 c	19.0 a	18.8 a	20.5 A	2.5 C	14.9 B
Cubenene	20.6 b	21.3 b	30.3 a	2.9 c	16.9 b	17.1 b	37.2 A	3.6 C	13.6 B
δ-Amorphene	52.3 ab	52.6 ab	62.0 a	5.8 b	56.5 ab	57.5 ab	92.9 A	8.0 C	42.5 B
Selina-3,7(11)-diene	38.3 ab	24.9 ab	78.1 a	4.6 b	36.5 ab	35.5 ab	74.9 A	7.2 C	26.8 B
Total sesquiterpenes	943.0 c	1373.9 ab	1219.3 bc	173.1 d	1247.2 bc	1680.1 a	2103.9 A	102.5 C	1111.9 B
***Oxygenated sesquiterpenes***									
α-Bisabolol	9.9 bc	35.6 a	10.7 bc	2.0 c	17.7 bc	25.5 ab	34.7 A	10.2 B	5.8 B
Caryophyllene oxide	72.7 a	59.3 a	68.3 a	19.5 b	52.7 a	55.4 a	90.6 A	33.2 B	40.1 B
Humulene epoxide II	19.7 a	17.5 a	13.5 a	3.6 b	18.8 a	18.7 a	31.2 A	6.1 B	8.6 B
*trans*-Longipinocarveol	38.2 a	30.1 a	31.6 a	6.4 b	23.7 a	24.9 a	56.8 A	12.6 B	8.1 B
Longifolenaldehyde	22.3 a	12.5 b	24.3 a	3.2 c	10.9 bc	11.5 b	38.0 A	4.4 B	0.0 C
Alloaromadendrene oxide	22.4 a	12.2 abc	23.5 a	2.9 c	14.8 ab	8.5 bc	32.1 A	7.3 B	2.8 B
Eudesm-7(11)-en-4-ol	14.6 a	14.0 a	12.6 a	2.6 b	17.6 a	17.9 a	26.9 A	5.1 B	7.6 B
Clovanediol	9.8 a	6.3 b	5.8 b	1.7 c	1.9 c	3.1 bc	13.7 A	0.6 B	0.0 B
Total oxygenated sesquiterpenes	209.7 a	187.4 a	190.3 a	42.0 b	158.1 a	165.5 a	323.9 A	79.6 B	73.0 B
***Triterpenes***									
Phytol	26.6 b	43.4 ab	33.6 b	40.7 ab	56.1 ab	74.4 a	115.0 A	14.4 B	8.0 B
α-Amyrin	5.8 b	4.9 b	4.9 b	9.2 a	4.4 b	4.5 b	13.7 A	2.0 B	1.2 B
Total triterpenes	32.2 b	48.2 ab	38.5 b	49.9 ab	60.5 ab	78.9 a	128.7 A	16.4 B	9.1 B
***Cannabinoids***									
Cannabidivarin	452.2 b	969.9 a	805.1 ab	18.2 c	858.0 ab	1128.6 a	1590.5 A	277.0 B	248.6 B
Cannabidiol (CBD)	15,814.2 c	22,329.8 bc	24,487.5 b	2573.8 d	25,254.3 b	35,575.9 a	31,019.6 A	16,122.7 B	15,875.5 B
Δ9-Tetrahydrocannabinol (THC)	688.8 c	819.9 c	936.5 bc	28.6 d	1153.6 ab	1283.9 a	1625.9 A	353.6 B	476.2 B
Cannabigerol (CBG)	1404.1 bc	972.6 c	893.8 c	6370.0 a	1660.5 bc	1820.8 b	4182.9 A	848.9 C	1529.1 B
Cannabinol	218.7 a	204.9 a	266.6 a	5.8 b	163.4 a	158.2 a	418.7 A	74.8 B	15.2 B
Total cannabinoids	18,577.9 c	25,297.2 bc	27,389.5 b	8996.5 d	29,089.8 b	39,967.4 a	38,837.4 A	17,677.0 B	18,144.6 B
***Tocopherols***									
γ-Tocopherol	80.2 ab	52.4 c	43.8 c	41.8 c	87.2 a	64.4 bc	113.9 A	55.3 B	15.7 C
α-Tocopherol	301.6 a	213.3 a	270.8 a	212.4 a	294.3 a	456.5 a	645.0 A	166.4 B	63.1 B
Total tocopherols	381.8 ab	265.7 ab	314.6 ab	254.2 b	381.5 ab	520.9 a	758.8 A	221.7 B	78.8 B
***Phytosterols***									
Campesterol	13.9 ab	16.8 ab	8.7 b	16.4 ab	21.2 a	19.9 a	37.1 A	6.2 B	5.2 B
γ-Sitosterol	14.0 a	11.9 a	5.6 a	11.4 a	12.8 a	12.2 a	24.7 A	6.1 B	3.2 B
Total phytosterols	27.9 ab	28.7 ab	14.3 b	27.8 ab	34.0 a	32.1 ab	61.8 A	12.3 B	8.4 B
Total phytochemicals	20,035.9 c	36,701.3 b	40,603.8 b	17,128.5 d	38,528.3 b	53,543.7 a	58,832.2 A	25,080.9 B	23,357.6 B
ABTS	139.2 d	162.5 c	211.2 a	139.9 d	160.0 c	171.8 b	199.5 B	218.8 A	74.0 C
DPPH	100.5 d	110.9 c	122.4 a	112.7 c	122.6 a	116.5 b	125.5 A	113.7 B	103.5 C

^1^ Data are expressed as follows: TPC, mg ferulic acid equivalents/g dry matter (d.m.); TFC, mg catechin equivalents/g d.m.; metabolite/class, μg/g d.m.; ABTS and DPPH radical scavenging activity, mg Trolox equivalent/g d.m. Different lower-case letters in the same row represent significant differences among genotypes (Tukey’s test *p* < 0.05), whereas different capital letters in the same row represent significant differences among years (Tukey’s test *p* < 0.05).

## Data Availability

Data is contained within the article or Supplementary Material.

## Supplementary Materials

The following supporting information can be downloaded at: https://www.mdpi.com/article/10.3390/ijms24108969/s1.

## References

[1] Simiyu D.C., Jang J.H., Lee O.R. (2022). Understanding *Cannabis sativa* L.: Current status of propagation, use, legalization, and haploid-inducer-mediated genetic engineering. Plants.

[2] Ren G., Zhang X., Li Y., Ridout K., Serrano-Serrano M.L., Yang Y., Liu A., Ravikanth G., Nawaz M.A., Mumtaz A.S. (2021). Large-scale whole-genome resequencing unravels the domestication history of *Cannabis sativa*. Sci. Adv..

[3] Small E. (2015). Evolution and classification of *Cannabis sativa* (marijuana, hemp) in relation to human utilization. Bot. Rev..

[4] Salentijn E.M., Petit J., Trindade L.M. (2019). The complex interactions between flowering behavior and fiber quality in hemp. Front. Plant Sci..

[5] Chandra S., Lata H., ElSohly M.A. (2017). Cannabis sativa L.-Botany and Biotechnology.

[6] Fournier G., Richez-Dumanois C., Duvezin J., Mathieu J.-P., Paris M. (1987). Identification of a new chemotype in *Cannabis sativa*: Cannabigerol-dominant plants, biogenetic and agronomic prospects. Planta Med..

[7] Mandolino G., Carboni A. (2004). Potential of marker-assisted selection in hemp genetic improvement. Euphytica.

[8] Plant Variety Catalogues, Databases & Information Systems. https://food.ec.europa.eu/plants/plant-reproductive-material/plant-variety-catalogues-databases-information-systems_en#agri-veg.

[9] Regulation (EU) 2021/2115 of the European Parliament and of the Council of 2 December 2021 Establishing Rules on Support for Strategic Plans to Be Drawn up by Member States under the Common Agricultural Policy (CAP Strategic Plans) and Financed by the European Agricultural Guarantee Fund (EAGF) and by the European Agricultural Fund for Rural Development (EAFRD) and Repealing Regulations (EU) No 1305/2013 and (EU) No 1307/2013. https://eur-lex.europa.eu/legal-content/EN/TXT/?uri=CELEX%3A32021R2115.

[10] Kaur N., Sharma L.K., Kelly-Begazo C., Tancig M., Brym Z. (2021). Uses of raw products obtained from hemp: Fiber, seed, and cannabinoids. UF/IFAS Ext..

[11] Ahmed A.T.M.F., Islam M.Z., Mahmud M.S., Sarker M.E., Islam M.R. (2022). Hemp as a potential raw material toward a sustainable world: A review. Heliyon.

[12] AL Ubeed H.M.S., Brennan C.S., Schanknecht E., Alsherbiny M.A., Saifullah M., Nguyen K., Vuong Q.V. (2022). Potential applications of hemp (*Cannabis sativa* L.) extracts and their phytochemicals as functional ingredients in food and medicinal supplements: A narrative review. Int. J. Food Sci. Technol..

[13] Frassinetti S., Moccia E., Caltavuturo L., Gabriele M., Longo V., Bellani L., Giorgi G., Giorgetti L. (2018). Nutraceutical potential of hemp (*Cannabis sativa* L.) seeds and sprouts. Food Chem..

[14] Calzolari D., Magagnini G., Lucini L., Grassi G., Appendino G.B., Amaducci S. (2017). High added-value compounds from *Cannabis* threshing residues. Ind. Crops Prod..

[15] Naeem M.Y., Corbo F., Crupi P., Clodoveo M.L. (2023). Hemp: An alternative source for various industries and an emerging tool for functional food and pharmaceutical sectors. Processes.

[16] Aliferis K.A., Bernard-Perron D. (2020). Cannabinomics: Application of metabolomics in *Cannabis* (*Cannabis sativa* L.) research and development. Front. Plant Sci..

[17] Cerrato A., Citti C., Cannazza G., Capriotti A.L., Cavaliere C., Grassi G., Marini F., Montone C.M., Paris R., Piovesana S. (2021). Phytocannabinomics: Untargeted metabolomics as a tool for cannabis chemovar differentiation. Talanta.

[18] Radwan M.M., Chandra S., Gul S., ElSohly M.A. (2021). Cannabinoids, phenolics, terpenes and alkaloids of *Cannabis*. Molecules.

[19] Booth J.K., Bohlmann J. (2019). Terpenes in *Cannabis sativa*–From plant genome to humans. Plant Sci..

[20] Russo E.B., Marcu J. (2017). Chapter three: Cannabis pharmacology: The usual suspects and a few promising leads. Adv. Pharmacol..

[21] Peng H., Shahidi F. (2021). Cannabis and Cannabis edibles: A review. J. Agric. Food Chem..

[22] Smith G.H., Roberts J.M., Pope T.W. (2018). Terpene based biopesticides as potential alternatives to synthetic insecticides for control of aphid pests on protected ornamentals. Crop. Prot..

[23] Masyita A., Sari R.M., Astuti A.D., Yasir B., Rumata N.R., Emran T.B., Firzan Nainu F., Simal-Gandara J. (2022). Terpenes and terpenoids as main bioactive compounds of essential oils, their roles in human health and potential application as natural food preservatives. Food Chem. X.

[24] Rahman M.M., Rahaman M.S., Islam M.R., Rahman F., Mithi F.M., Alqahtani T., Almikhlafi M.A., Alghamdi S.Q., Alruwaili A.S., Hossain M.S. (2021). Role of phenolic compounds in human disease: Current knowledge and future prospects. Molecules.

[25] Bautista J.L., Yu S., Tian L. (2021). Flavonoids in *Cannabis sativa*: Biosynthesis, bioactivities, and biotechnology. ACS Omega.

[26] André A., Leupin M., Kneubühl M., Pedan V., Chetschik I. (2020). Evolution of the polyphenol and terpene content, antioxidant activity and plant morphology of eight different fiber-type cultivars of *Cannabis sativa* L. cultivated at three sowing densities. Plants.

[27] Ingallina C., Sobolev A.P., Circi S., Spano M., Fraschetti C., Filippi A., Di Sotto A., Di Giacomo S., Mazzoccanti G., Gasparrini F. (2020). *Cannabis sativa* L. inflorescences from monoecious cultivars grown in central Italy: An untargeted chemical characterization from early flowering to ripening. Molecules.

[28] Milay L., Berman P., Shapira A., Guberman O., Meiri D. (2020). Metabolic profiling of *Cannabis* secondary metabolites for evaluation of optimal postharvest storage conditions. Front. Plant Sci..

[29] Spano M., Di Matteo G., Ingallina C., Botta B., Quaglio D., Ghirga F., Balducci S., Cammarone S., Campiglia E., Giusti A.M. (2021). A multimethodological characterization of *Cannabis sativa* L. inflorescences from seven dioecious cultivars grown in Italy: The effect of different harvesting stages. Molecules.

[30] Spano M., Di Matteo G., Ingallina C., Sobolev A.P., Giusti A.M., Vinci G., Cammarone S., Tortora C., Lamelza L., Prencipe S.A. (2022). Industrial hemp *(Cannabis sativa* L.) inflorescences as novel food: The effect of different agronomical practices on chemical profile. Foods.

[31] Irakli M., Tsaliki E., Kalivas A., Kleisiaris F., Sarrou E., Cook C.M. (2019). Effect οf genotype and growing year on the nutritional, phytochemical, and antioxidant properties of industrial hemp (*Cannabis sativa* L.) seeds. Antioxidants.

[32] Menga V., Garofalo C., Suriano S., Beleggia R., Colecchia S.A., Perrone D., Montanari M., Pecchioni N., Fares C. (2022). Phenolic acid composition and antioxidant activity of whole and defatted seeds of Italian hemp cultivars: A two-year case study. Agriculture.

[33] Pieracci Y., Ascrizzi R., Terreni V., Pistelli L., Flamini G., Bassolino L., Fulvio F., Montanari M., Paris R. (2021). Essential oil of *Cannabis sativa* L: Comparison of yield and chemical composition of 11 hemp genotypes. Molecules.

[34] Ferrante C., Recinella L., Ronci M., Menghini L., Brunetti L., Chiavaroli A., Leone S., Di Iorio L., Carradori S., Tirillini B. (2019). Multiple pharmacognostic characterization on hemp commercial cultivars: Focus on inflorescence water extract activity. Food Chem. Toxicol..

[35] Izzo L., Castaldo L., Narváez A., Graziani G., Gaspari A., Rodríguez-Carrasco Y., Ritieni A. (2020). Analysis of phenolic compounds in commercial *Cannabis sativa* L. inflorescences using UHPLC-Q-Orbitrap HRMS. Molecules.

[36] Šeruga M., Novak I., Jakobek L. (2011). Determination of polyphenols content and antioxidant activity of some red wines by differential pulse voltammetry, HPLC and spectrophotometric methods. Food Chem..

[37] Petruccelli R., Ieri F., Ciaccheri L., Bonetti A. (2018). Polyphenolic profiling and chemometric analysis of leaves from Italian *Ficus carica* L. varieties. Polyphenol compounds in common fig. Eur. J. Hortic. Sci..

[38] Luaces P., Pascual M., Pérez A.G., Sanz C. (2021). An easy-to-use procedure for the measurement of total phenolic compounds in olive fruit. Antioxidants.

[39] Nissen L., Zatta A., Stefanini I., Grandi S., Sgorbati B., Biavati B., Monti A. (2010). Characterization and antimicrobial activity of essential oils of industrial hemp varieties (*Cannabis sativa* L.). Fitoterapia.

[40] Barčauskaitė K., Bakšinskaitė A., Szumny A., Tilvikienė V. (2022). Variation of secondary metabolites in *Cannabis sativa* L. inflorescences under applied agrotechnological measures. Ind. Crops Prod..

[41] Pieracci Y., Fulvio F., Isca V., Pistelli L., Bassolino L., Montanari M., Moschella A., Flamini G., Paris R. (2023). The phenological stage of hemp inflorescences affects essential oil yield and its chemical composition. Ind. Crops Prod..

[42] Pacifico D., Miselli F., Carboni A., Moschella A., Mandolino G. (2008). Time course of cannabinoid accumulation and chemotype development during the growth of *Cannabis sativa* L. Euphytica.

[43] Floegel A., Kim D.-O., Chung S.-J., Koo S.I., Chun O.K. (2011). Comparison of ABTS/DPPH assays to measure antioxidant capacity in popular antioxidant-rich US foods. J. Food Compost. Anal..

[44] Bettaieb I., Zakhama N., Aidi Wannes W., Kchouk M.E., Marzouk B. (2009). Water deficit effects on *Salvia officinalis* fatty acids and essential oils composition. Sci. Hortic..

[45] Savoi S., Wong D.C., Arapitsas P., Miculan M., Bucchetti B., Peterlunger E., Fait A., Mattivi F., Castellarin S.D. (2016). Transcriptome and metabolite profiling reveals that prolonged drought modulates the phenylpropanoid and terpenoid pathway in white grapes (*Vitis vinifera* L.). BMC Plant Biol..

[46] Caplan D., Dixon M., Zheng Y. (2019). Increasing inflorescence dry weight and cannabinoid content in medical cannabis using controlled drought stress. Hort. Sci..

[47] Campbell B.J., Berrada A.F., Hudalla C., Amaducci S., McKay J.K. (2019). Genotype × environment interactions of industrial hemp cultivars highlight diverse responses to environmental factors. Agrosyst. Geosci. Environ..

[48] Kopustinskiene D.M., Masteikova R., Lazauskas R., Bernatoniene J. (2022). *Cannabis sativa* L. bioactive compounds and their protective role in oxidative stress and inflammation. Antioxidants.

[49] Hanuš L.O., Hod Y. (2020). Terpenes/terpenoids in *Cannabis*: Are they important?. Med. Cannabis Cannabinoids.

[50] Malherbe C.J., De Beer D., Joubert E. (2012). Development of on-line high performance liquid chromatography (HPLC)-biochemical detection methods as tools in the identification of bioactives. Int. J. Mol. Sci..

[51] Mishchenko S., Mokher J., Laiko I., Burbulis N., Kyrychenko H., Dudukova S. (2017). Phenological growth stages of hemp (*Cannabis sativa* L.): Codification and description according to the BBCH scale. Žemės Ūkio Moksl..

[52] Quitadamo F., De Simone V., Beleggia R., Trono D. (2021). Chitosan-induced activation of the antioxidant defense system counteracts the adverse effects of salinity in durum wheat. Plants.

[53] Fares C., Menga V. (2012). Effects of toasting on the carbohydrate profile and antioxidant properties of chickpea (*Cicer arietinum* L.) flour added to durum wheat pasta. Food Chem..

[54] Benthin B., Danz H., Hamburger M. (1999). Pressurized liquid extraction of medicinal plants. J. Chromatogr. A.

[55] Adams R.P. (2007). Identification of Essential Oil Components by Gas Chromatography/Mass Spectrometry.

[56] Linstorm P. (1998). NIST Chemistry WebBook, NIST Standard Reference Database Number 69. J. Phys. Chem. Ref. Data, Monograph.

[57] Iannucci A., Suriano S., Cancellaro S., Trono D. (2022). Anthocyanin profile and main antioxidants in pigmented wheat grains and related millstream fractions. Cereal Chem..

[58] Hossain M.K., Jena K.K., Bhuiyan M.A., Wickneswari R. (2016). Association between QTLs and morphological traits toward sheath blight resistance in rice (*Oryza sativa* L.). Breed. Sci..

[59] Peng J., Fan M., An C., Ni F., Huang W., Luo J. (2022). A narrative review of molecular mechanism and therapeutic effect of cannabidiol (CBD). Basic Clin. Pharmacol. Toxicol..

[60] Fernandes E.S., Passos G., Medeiros R., Da Cunha F., Ferreira J., Campos M., Pianowski L., Calixto J. (2007). Anti-inflammatory effects of compounds alpha-humulene and (-)-trans-caryophyllene isolated from the essential oil of Cordia verbenacea. Eur. J. Pharmacol..

[61] Bahi A., Al Mansouri S., Al Memari E., Al Ameri M., Nurulain S.M., Ojha S. (2014). b-Caryophyllene, a CB2 receptor agonist produces multiple behavioral changes relevant to anxiety and depression in mice. Physiol. Behav..

[62] Jastrząb A., Jarocka-Karpowicz I., Skrzydlewska E. (2022). The origin and biomedical relevance of cannabigerol. Int. J. Mol. Sci..

