# Supplementary material for: Effect of Genotype, Year, and Their Interaction on the Accumulation of Bioactive Compounds and the Antioxidant Activity in Industrial Hemp (Cannabis sativa L.) Inflorescences
Source: Int J Mol Sci. 2023 May 18;24(10):8969. doi: 10.3390/ijms24108969 (PMC10219208; doi:10.3390/ijms24108969)
Supplement: Supplementary file 1 [file ijms-24-08969-s001.zip › Supplementary material/Table S1.pdf]

Table S1. Percentage of contribution to the variability for genotype (G), year (Y) and genotype × year (G × Y) interaction for all the traits analyzed in the inflorescences of the six hemp genotypes grown for three consecutive years (2018-2020).

**Trait**	**G (%)**	**Y (%)**	**G × Y (%)**
TPC	12.8	67.0	17.6
TFC	2.3	88.5	7.8
Phenolic acids			
Gallic acid	10.1	55.8	31.4
Vanillic acid	14.4	43.3	32.7
p-Hydroxybenzoic acid	4.9	82.4	8.5
Caffeic acid	12.2	61.7	23.6
p-Coumaric acid	12.4	70.0	13.8
Total phenolic acids	6.4	71.6	20.5
Flavonoids			
Epicatechin	14.6	52.7	29.5
Catechin	31.3	20.3	42.2
Orientin	2.6	93.7	2.3
Rutin	6.4	86.1	5.7
Vitexin	13.8	66.7	15.0
Naringenin	7.6	56.3	32.4
Total flavonoids	6.5	88.5	3.7
Monoterpenes			
α-Pinene	20.9	47.0	23.1
β-Pinene	25.8	34.0	27.2
β-Myrcene	18.9	48.8	17.0
3-Carene	20.4	43.6	17.7
α-Phellandrene	18.9	41.4	21.5
Limonene	29.4	31.0	27.9
trans-Ocimene	19.6	43.7	26.3
γ-Terpinene	28.4	23.0	43.7
Total monoterpenes	18.9	51.1	21.8
Oxygenated monoterpenes			
Eucalyptol	23.7	24.6	46.8
trans-Sabinene hydrate	24.5	34.8	27.1
Fenchol	13.7	43.4	27.3
trans-2-Pinanol	9.6	32.7	17.8
Terpinen-4-ol	21.2	52.3	14.5
p-Cymen-8-ol	17.0	25.5	35.5
α-Terpineol	23.0	45.4	18.7
Total oxygenated monoterpenes	19.6	50.1	20.7
Sesquiterpenes			
α-Ylangene	11.1	66.9	15.5
Isocaryophyllene	13.7	55.4	15.4
β-Caryophyllene	22.0	59.8	13.8
α-Humulene	24.9	54.3	13.8
Aromadendrene	17.6	56.3	20.1

β-Himachalene	25.2	37.8	29.1
4,11-Selinadiene	14.1	54.7	23.9
β-Selinene	18.4	38.4	30.0
β-Cadinene	20.9	54.1	15.6
α-Selinene	16.0	42.7	32.3
β-Curcumene	4.4	66.2	6.4
(*E*)-γ-Bisabolene	26.1	39.9	22.8
Cubenene	16.7	50.4	28.9
δ-Amorphene	11.7	39.0	18.4
Selina-3,7(11)-diene	14.7	24.5	29.0
Total sesquiterpenes	20.3	61.0	13.3
Oxygenated sesquiterpenes			
α-Bisabolol	24.1	31.6	25.9
Caryophyllene oxide	18.8	41.8	27.9
Humulene epoxide II	14.6	59.6	16.5
trans-Longipinocarveol	12.0	59.7	18.6
Longifolenaldehyde	11.8	65.7	17.8
Alloaromadendrene oxide	15.5	48.3	22.8
Eudesm-7(11)-en-4-ol	15.5	56.2	15.6
Clovanediol	12.9	62.2	17.9
Total oxygenated sesquiterpenes	13.6	61.3	18.8
Triterpenes			
Phytol	7.5	73.3	5.7
α-Amyrin	6.0	72.7	13.5
Total triterpenes	5.9	76.5	4.5
Cannabinoids			
Cannabidivarin	18.7	53.6	20.2
Cannabidiol (CBD)	44.6	22.0	24.5
Δ9-Tetrahydrocannabinol (THC)	26.4	52.9	16.6
Cannabigerol (CBG)	46.2	26.5	24.8
Cannabinol	12.0	56.8	23.6
Total cannabinoids	34.0	36.5	21.4
Tocopherols			
γ-Tocopherol	11.8	63.8	17.6
α-Tocopherol	6.8	65.3	3.9
Total tocopherols	6.6	70.1	4.1
Phytosterols			
Campesterol	5.9	76.4	7.6
γ-Sitosterol	4.3	54.8	14.4
Total phytosterols	4.8	71.1	10.0
Total phytochemicals	26.2	55.5	13.3
ABTS	10.2	71.9	17.5
DPPH	8.0	11.2	80.1

TPC, total phenolic content; TFC, total flavonoid content; ABTS, 2,2-azinobis-(3-ethylbenzothiazoline-6-sulphonic acid) radical scavenging activity; DPPH, 2,2-diphenyl-1-picrylhydrazyl radical scavenging activity.
