# Supplementary material for: Effect of Genotype, Year, and Their Interaction on the Accumulation of Bioactive Compounds and the Antioxidant Activity in Industrial Hemp (Cannabis sativa L.) Inflorescences
Source: Int J Mol Sci. 2023 May 18;24(10):8969. doi: 10.3390/ijms24108969 (PMC10219208; doi:10.3390/ijms24108969)
Supplement: Supplementary file 1 [file ijms-24-08969-s001.zip › Supplementary material/Table S2.pdf]

Table S2. *Eigenvalues*, *eigenvectors* and percentage of variation explained by the first eight axes for all the traits analyzed in the inflorescences of the six hemp genotypes grown in three consecutive years (2018-2020).

	**Principal Component Axis**							
	**1**	**2**	**3**	**4**	**5**	**6**	**7**	**8**
Eigenvalues	*37.74*	*7.99*	*4.94*	*3.72*	*2.02*	*1.92*	*1.56*	*0.99*
PC variation (%)	59.0	12.5	7.7	5.8	3.1	3.0	2.4	1.5
Cumulative variation (%)	59.0	71.5	79.2	85.0	88.1	91.1	93.6	95.1
**Trait**	***Eigenvectors***							
TPC	0.114	0.160	-0.150	0.163	-0.004	-0.029	-0.114	-0.117
TFC	0.151	0.058	-0.040	-0.098	0.145	0.019	-0.019	-0.016
Gallic acid	0.099	0.160	-0.126	0.124	0.067	-0.124	-0.080	0.288
Vanillic acid	-0.039	0.214	0.132	0.214	0.071	0.267	0.049	0.198
p-Hydroxybenzoic acid	-0.074	0.248	0.089	0.188	-0.087	-0.104	0.121	0.160
Caffeic acid	-0.060	0.212	0.103	0.260	0.051	0.194	-0.019	-0.100
p-Coumaric acid	0.092	0.254	0.065	-0.079	0.055	0.164	0.143	-0.072
Epicatechin	0.010	0.247	0.112	0.177	0.194	0.211	-0.055	-0.206
Catechin	-0.021	0.123	0.231	0.237	0.308	0.228	0.093	-0.060
Orientin	0.143	0.133	-0.081	-0.060	0.081	0.086	-0.053	-0.070
Rutin	0.141	0.018	-0.081	-0.110	0.221	0.095	-0.061	-0.126
Vitexin	-0.034	0.268	0.045	0.198	-0.059	-0.162	0.200	0.181
Naringenin	0.070	0.239	0.059	-0.113	-0.142	0.028	0.202	-0.097
α-Pinene	0.144	-0.015	0.170	-0.082	-0.018	0.052	-0.013	0.046
β-Pinene	0.121	-0.043	0.172	-0.106	-0.085	0.062	-0.191	0.080
β-Myrcene	0.146	-0.062	-0.018	0.085	0.013	0.185	0.010	0.105
3-Carene	0.145	-0.114	0.025	0.092	0.072	-0.031	0.082	-0.032
α-Phellandrene	0.147	-0.097	0.049	0.090	0.118	-0.088	0.070	-0.044
Limonene	0.120	-0.126	-0.039	0.208	0.158	0.062	0.024	0.136
trans-Ocimene	0.119	-0.147	0.078	-0.035	-0.103	0.236	0.174	0.158
γ-Terpinene	0.074	-0.219	0.006	0.231	0.199	0.014	0.161	0.185
Eucalyptol	0.094	-0.167	0.176	0.002	0.085	-0.055	0.368	-0.080
trans-Sabinene hydrate	0.112	-0.191	-0.057	0.150	0.048	0.048	0.212	-0.024
Fenchol	0.125	0.038	-0.165	0.159	0.032	-0.091	-0.279	-0.116
trans-2-Pinanol	0.123	0.060	-0.148	0.133	-0.054	-0.024	-0.337	0.141
Terpinen-4-ol	0.142	-0.085	-0.098	0.091	0.072	0.124	0.068	-0.036
p-Cymen-8-ol	0.115	0.054	0.199	-0.124	0.174	-0.215	0.059	-0.172
α-Terpineol	0.137	-0.087	-0.116	0.156	0.061	-0.035	-0.039	-0.064
α-Ylangene	0.156	0.014	0.047	-0.045	-0.072	0.094	-0.096	0.033
Isocaryophyllene	0.147	-0.032	0.043	-0.109	-0.084	0.075	-0.091	-0.149
β-Caryophyllene	0.144	-0.115	-0.064	0.064	-0.032	0.091	-0.034	-0.090
α-Humulene	0.136	-0.136	-0.102	0.086	-0.054	0.068	0.003	-0.138
Aromadendrene	0.151	-0.035	0.088	-0.052	-0.023	-0.050	0.062	-0.105

β-Himachalene	0.134	0.012	0.209	-0.083	0.093	-0.069	-0.095	-0.073
4,11-Selinadiene	0.155	-0.032	0.093	0.018	0.050	-0.043	-0.111	0.042
β-Selinene	0.137	-0.086	0.171	-0.025	0.098	-0.111	-0.122	-0.027
β-Cadinene	0.151	-0.073	-0.054	0.109	-0.033	0.036	-0.025	0.099
α-Selinene	0.144	-0.037	0.157	-0.013	0.104	-0.115	-0.130	-0.016
β-Curcumene	0.139	0.088	-0.146	-0.049	0.116	-0.001	-0.018	-0.159
(E)-γ-Bisabolene	0.130	-0.164	-0.022	0.160	-0.013	0.007	0.005	0.103
Cubenene	0.150	-0.027	0.158	-0.045	-0.017	-0.008	-0.036	0.041
δ-Amorphene	0.153	-0.074	0.079	0.025	-0.027	0.033	0.002	0.083
Selina-3,7(11)-diene	0.132	-0.015	0.205	-0.026	0.094	-0.054	-0.207	0.097
α-Bisabolol	0.117	0.060	-0.078	0.052	-0.199	-0.121	0.299	-0.328
Caryophyllene oxide	0.140	0.022	0.118	-0.110	-0.186	0.049	0.050	0.071
Humuleneepoxide II	0.154	0.012	-0.021	0.016	-0.159	0.040	0.077	0.137
trans-Longipinocarveol	0.148	0.082	0.063	-0.071	-0.153	0.049	0.078	0.079
Longifolenaldehyde	0.143	0.103	0.083	-0.116	-0.054	0.106	-0.088	0.051
Alloaromadendrene oxide	0.136	0.088	0.135	-0.115	-0.066	0.051	-0.046	0.107
Eudesm-7(11)-en-4-ol	0.156	0.006	-0.040	0.068	-0.125	0.020	0.004	0.100
Clovanediol	0.135	0.090	0.056	-0.166	-0.125	0.171	0.106	0.101
Phytol	0.126	0.093	-0.241	-0.018	0.086	0.020	0.022	-0.082
α-Amyrin	0.099	0.121	-0.136	-0.257	0.221	-0.006	0.139	0.142
Cannabidivarin	0.149	0.039	-0.062	0.081	-0.089	-0.059	0.045	-0.250
Cannabidiol (CBD)	0.127	0.000	0.035	0.189	-0.158	-0.194	0.180	-0.061
Δ9-Tetrahydrocannabinol (THC)	0.155	0.001	-0.010	0.105	-0.085	-0.042	0.009	-0.020
Cannabigerol (CBG)	0.022	0.051	-0.188	-0.284	0.421	-0.023	0.182	0.245
Cannabinol	0.149	0.091	0.102	-0.037	-0.114	-0.028	-0.005	0.009
γ-Tocopherol	0.114	0.152	-0.164	0.055	-0.222	-0.018	-0.011	0.200
α-Tocopherol	0.135	0.134	-0.147	-0.041	0.014	0.007	0.007	0.013
Campesterol	0.123	0.084	-0.257	-0.053	0.045	-0.013	0.065	0.019
γ-Sitosterol	0.112	0.106	-0.242	-0.083	0.001	-0.067	0.106	0.028
ABTS	0.048	0.280	0.131	0.090	0.081	-0.181	-0.079	-0.097
DPPH	0.077	0.053	0.132	0.053	0.105	-0.545	0.044	0.171
