# Supplementary material for: Effect of Genotype, Year, and Their Interaction on the Accumulation of Bioactive Compounds and the Antioxidant Activity in Industrial Hemp (Cannabis sativa L.) Inflorescences
Source: Int J Mol Sci. 2023 May 18;24(10):8969. doi: 10.3390/ijms24108969 (PMC10219208; doi:10.3390/ijms24108969)
Supplement: Supplementary file 1 [file ijms-24-08969-s001.zip › Supplementary material/Table S3.pdf]

Table S3. GC/MS identification information.

Metabolite	Formula	RT[a]	LRI[b]	Identification[c]
α-Pinene	$C_{10}H_{16}$	8.29	935	MS, Std, RI
β-Pinene	$C_{10}H_{16}$	9.24	980	MS, Std, RI
β-Myrcene	$C_{10}H_{16}$	9.47	990	MS, RI
3-Carene	$C_{10}H_{16}$	9.89	1004	MS, Std, RI
α-Phellandrene	$C_{10}H_{16}$	10.15	1009	MS, Std, RI
Limonene	$C_{10}H_{16}$	10.46	1015	MS, Std, RI
trans-Ocimene	$C_{10}H_{16}$	10.88	1023	MS, Std, RI
Eucalyptol	$C_{10}H_{18}O$	11.16	1028	MS, RI
γ-Terpinene	$C_{10}H_{16}$	12.01	1044	MS, Std, RI
trans-Sabinene hydrate	$C_{10}H_{18}O$	12.54	1054	MS, RI
Fenchol	$C_{10}H_{18}O$	12.69	1115	MS, Std, RI
trans-2-Pinanol	$C_{10}H_{18}O$	12.91	1124	MS, RI
Terpinen-4-ol	$C_{10}H_{18}O$	14.41	1179	MS, RI
p-Cymenol	$C_{10}H_{18}O$	14.59	1186	MS, RI
α-Terpineol	$C_{10}H_{18}O$	14.77	1192	MS,Std, RI
α-Ylangene	$C_{15}H_{24}$	19.71	1379	MS, RI
Isocaryophyllene	$C_{15}H_{24}$	20.56	1412	MS, RI
β-Caryophyllene	$C_{15}H_{24}$	21.23	1439	MS, Std, RI
α-Humulene	$C_{15}H_{24}$	22.01	1470	MS, Std, RI
Aromadendrene	$C_{15}H_{24}$	22.28	1481	MS, Std, RI
β-Himachalene	$C_{15}H_{24}$	22.33	1483	MS, RI
4,11-Selinadiene	$C_{15}H_{24}$	22.40	1486	MS, RI
β-Selinene	$C_{15}H_{24}$	22.48	1489	MS, RI
β-Cadinene	$C_{15}H_{24}$	22.53	1491	MS, RI
α-Selinene	$C_{15}H_{24}$	22.76	1501	MS, RI
β-Curcumene	$C_{15}H_{24}$	23.05	1513	MS, RI
(E)-γ-Bisabolene	$C_{15}H_{24}$	23.33	1525	MS, RI
Cubenene	$C_{15}H_{24}$	23.43	1532	MS, RI
δ-Amorphene	$C_{15}H_{24}$	23.70	1541	MS, RI
Selina-3,7(11)-diene	$C_{15}H_{24}$	23.82	1546	MS, RI
Caryophyllene oxide	$C_{15}H_{24}O$	24.80	1588	MS, RI
Humulene epoxide II	$C_{15}H_{24}O$	25.69	1614	MS, RI
trans-Longipinocarveol	$C_{15}H_{24}O$	25.90	1619	MS, RI
Longifolenaldehyde	$C_{15}H_{24}O$	26.54	1633	MS, RI
Alloaromadendrene oxide	$C_{15}H_{24}O$	26.83	1640	MS, RI
α-Bisabolol	$C_{15}H_{26}O$	28.18	1671	MS, RI
Eudesm-7(11)-en-4-ol	$C_{15}H_{26}O$	28.40	1676	MS, RI
Clovanediol	$C_{15}H_{26}O_2$	31,13	1881	MS, RI
Phytol	$C_{20}H_{40}O$	35.80	2081	MS, RI

Cannabidivarin	$C_{19}H_{26}O_2$	37.18	2214	MS, RI
Cannabidiol (CBD)	$C_{21}H_{30}O_2$	39.91	2425	MS, RI
Δ9-Tetrahydrocannabinol (THC)	$C_{21}H_{30}O_2$	40.79	2467	MS, RI
Cannabigerol (CBG)	$C_{21}H_{30}O_2$	41.34	2493	MS, RI
Cannabinol	$C_{21}H_{26}O_2$	41,70	2511	MS, RI
γ-Tocopherol	$C_{28}H_{48}O_2$	47.78	3026	MS, Std
α-Tocopherol	$C_{29}H_{50}O_2$	49.44	3065	MS, Std
Campesterol	$C_{28}H_{48}O$	51.84	3215	MS, Std
γ-Sitosterol	$C_{29}H_{50}O$	54.40	3256	MS, Std
α-Amyrin	$C_{30}H_{50}O$	55.49	3274	MS, Std

[a]RT, Retention Time (min), compounds are listed in order of their elution from HP-5MS column.
[b]LRI, Linear Retention Index on HP-5MS column experimentally determined using homologous series of C_8-C_{34} alkanes.
[c]Identification methods: MS, comparison of mass spectra with NIST11; Std, comparison of mass spectra with authentic standard compound; RI, comparison of experimental Retention Index with those reported in [55; 56].
